# Take one step backward to move forward: Assessment of genetic diversity and population structure of captive Asian woolly-necked storks (*Ciconia episcopus*)

**DOI:** 10.1371/journal.pone.0223726

**Published:** 2019-10-10

**Authors:** Kornsuang Jangtarwan, Tassika Koomgun, Tulyawat Prasongmaneerut, Ratchaphol Thongchum, Worapong Singchat, Panupong Tawichasri, Toshiharu Fukayama, Siwapech Sillapaprayoon, Ekaphan Kraichak, Narongrit Muangmai, Sudarath Baicharoen, Chainarong Punkong, Surin Peyachoknagul, Prateep Duengkae, Kornsorn Srikulnath

**Affiliations:** 1 Laboratory of Animal Cytogenetics and Comparative Genomics (ACCG), Department of Genetics, Faculty of Science, Kasetsart University, Bangkok, Thailand; 2 Special Research Unit for Wildlife Genomics, Department of Forest Biology, Faculty of Forestry, Kasetsart University, Chatuchak, Bangkok, Thailand; 3 Department of Botany, Faculty of Science, Kasetsart University, Bangkok, Thailand; 4 Department of Fishery Biology, Faculty of Fisheries, Kasetsart University, Bangkok, Thailand; 5 Bureau of Research and Conservation, The Zoological Park Organization (ZPO), Bangkok, Thailand; 6 Khao Kheow Open Zoo, Chonburi, Thailand; 7 Center for Advanced Studies in Tropical Natural Resources (CASTNAR), National Research University-Kasetsart University (NRU-KU), Kasetsart University, Bangkok, Thailand; 8 Center of Excellence on Agricultural Biotechnology (AG-BIO/PERDO-CHE), Bangkok, Thailand; 9 Omics Center for Agriculture, Bioresources, Food and Health, Kasetsart University (OmiKU), Bangkok, Thailand; SOUTHWEST UNIVERSITY, CHINA

## Abstract

The fragmentation of habitats and hunting have impacted the Asian woolly-necked stork (*Ciconia episcopus*), leading to a serious risk of extinction in Thailand. Programs of active captive breeding, together with careful genetic monitoring, can play an important role in facilitating the creation of source populations with genetic variability to aid the recovery of endangered species. Here, the genetic diversity and population structure of 86 Asian woolly-necked storks from three captive breeding programs [Khao Kheow Open Zoo (KKOZ) comprising 68 individuals, Nakhon Ratchasima Zoo (NRZ) comprising 16 individuals, and Dusit Zoo (DSZ) comprising 2 individuals] were analyzed using 13 microsatellite loci, to aid effective conservation management. Inbreeding and an extremely low effective population size (*N*_*e*_) were found in the KKOZ population, suggesting that deleterious genetic issues had resulted from multiple generations held in captivity. By contrast, a recent demographic bottleneck was observed in the population at NRZ, where the ratio of *N*_*e*_ to abundance (N) was greater than 1. Clustering analysis also showed that one subdivision of the KKOZ population shared allelic variability with the NRZ population. This suggests that genetic drift, with a possible recent and mixed origin, occurred in the initial NRZ population, indicating historical transfer between captivities. These captive stork populations require improved genetic variability and a greater population size, which could be achieved by choosing low-related individuals for future transfers to increase the adaptive potential of reintroduced populations. Forward-in-time simulations such as those described herein constitute the first step in establishing an appropriate source population using a scientifically managed perspective for an *in situ* and *ex situ* conservation program in Thailand.

## Introduction

The Asian woolly-necked stork (*Ciconia episcopus*) is a large endangered species on the IUCN Red List [1]. The birds mainly inhabit wetlands such as flood plains, rivers, ponds, swamps, tidal mudflats, cultivated fields, and even manmade tanks in tropical areas of Africa and Asia [2,3]. Historically, the Asian woolly-necked storks in Thailand have inhabited Khao Ang Rue Nai Wildlife Sanctuary (13°24’58.9278” and 101°56’26.793”), Ta Phraya National Park (14°7’43.5828” and 102°34’56.0706”), Songkhla Lake Wildlife Area (7°27’48.279” and 100°24’44.5608”) and Thale Noi Waterfowl Reserve (7°46’41.7504” and 100°7’22.0206”), with 30–35 storks recorded annually during 1980–2007, prior to a sharp decline in population during the late 2000s, which resulted from hunting, environmental pollution, and habitat fragmentation [4]. Only one bird was recorded in 2007 in the Nampad Wildlife Sanctuary (17°49’46.3” and 100°48’15.5”) [5]. This finding is very serious, implying a more imminent threat of extinction of the Asian woolly-necked stork in Thailand than in India and Nepal [6–8]. The reintroduction of captive-bred individuals and *in situ*/*ex situ* management are thus necessary to recover endangered populations of the Asian woolly-necked stork, processes entailing decisions at a national policy level. Zoos have played an important role in the conservation of endangered species through scientific research and public education, preservation of genetic diversity, management policies that support increasing population sizes, and the reintroduction of captive-bred populations into their natural habitat [9,10]. To date, only three captive breeding programs in Thailand (Khao Kheow Open Zoo: KKOZ, Nakhon Ratchasima Zoo: NRZ, and Dusit Zoo: DSZ), totaling 86 storks, have successfully contributed to the reinforcement and re-establishment of decimated populations. Unfortunately, the existing captive breeding programs were begun without genetic background information of founders. Research on the conservation genetics of these populations is urgently required.

Captive breeding programs consist of the fundamental processes of the management of mating, and offer viability for minimal long-term *in situ*/*ex situ* management and the recovery of wild endangered populations [11,12]. Both ecological and demographic factors influence the ability of captive and reintroduced populations to adapt to future environmental change, but their adaptive potential is also driven by their genetic diversity [13–16]. Loss of genetic variation results in small captive populations and inbreeding depression, leading to decline of fitness and its consequences for populations and species [11]. Thus, increased levels of genetic diversity achieved through a minimized relatedness approach increases the possibility of re-establishing a self-sustaining population, which is necessary for future long-term management strategies [11,17]. This approach is also a useful way to screen candidate individuals and preferentially remove those whose allelic profiles are over-represented in the population [18,19]. Having first recognized the importance of this issue, and with the aim of optimizing the main future genetic goals of a program of reintroduction into the wild, we first determined the genetic diversity and population structure in the three captive stork populations (KKOZ, NRZ, and DSZ) in Thailand using microsatellites. Second, we examined the breeding strategy for the representation of genetic resources, using effective population size (*N*_*e*_) as one of the most important parameters with which to estimate currently captive populations. It is necessary to first take one-step backward before moving forward to assess the management of captive breeding programs. We conclude by making recommendations for the long-term genetic management of captive populations to maintain/increase adaptive potential. This study represents the first genetic assessment of an ongoing captive breeding and reintroduction program for the Asian woolly-necked stork. This bird is under serious threat of extinction in Thailand. Previous studies indicated a relatively high level of genetic diversity in both wild and captive populations of Oriental white stork (*C*. *boyciana*) in East Asia and Russia [20–23].

## Materials and methods

### Specimen collection and DNA extraction

Eighty-six Asian woolly-necked storks were captured from three captive breeding programs in Thailand (KKOZ, NRZ, and DSZ). This population numbers all individuals remaining in Thailand. The DSZ population comprised just two storks, and human-mediated rotation of mating pairs was not implemented in the KKOZ and NRZ populations. A blood sample was collected and all birds were released immediately in each captive breeding area. Blood samples were collected from the ventral tail vein using a 24-gauge needle attached to a 3-ml disposable syringe containing 10 mM ethylenediaminetetraacetic acid (EDTA). Whole genomic DNA was extracted following the standard salting-out protocol as described previously by Supikamolseni et al. [24] and used as templates for microsatellite genotyping. Detailed information on the sample individuals is presented in S1 Table. The sex of each individual was identified by morphological observation and molecular sexing [25,26]. The research was conducted under the authority of the Ministry of Natural Resources and Environment, Thailand. Animal care and all experimental procedures were approved by the Animal Experiment Committee, Zoological Park Organization under the Royal Patronage of His Majesty the King (ZPO) (approval no. 2560096003004) and conducted according to the Regulations on Animal Experiments at ZPO and Kasetsart University.

### Microsatellite genotyping

All 13 microsatellite primer sets were taken from Shephard et al. [27], Wang et al. [28], Huang and Zhou [29], Turjeman [30] and were developed originally from the white stork (*C*. *ciconia*) and oriental white stork (*C*. *boyciana*) (S2 Table). The 5'-end of the forward primer of each set of primers was labeled with fluorescent dye (6-FAM or HEX, Macrogen Inc., Seoul, Korea). PCR amplification was performed using 15 μl of 1× ThermoPol buffer containing 1.5 mM MgCl_2_, 0.2 mM dNTPs, 5.0 μM primers, 0.5 U of *Taq* polymerase (Apsalagen Co. Ltd., Bangkok, Thailand), and 25 ng of genomic DNA. The PCR conditions were as follows: initial denaturation at 94°C for 3 min, followed by 35 cycles of 94°C for 30 s, 50°C for 30 s, 72°C for 1 min with a final extension at 72°C for 7 min. The PCR products were firstly detected by electrophoresis on 1% agarose gels. Fluorescent DNA fragment length analysis was subsequently performed using 23 ABI 3730XLs automatic sequencer (Applied Biosystems, Foster City, United States) at the DNA sequencing service of Macrogen Inc., and allelic sizes were determined using Peak Scanner version 1.0 (Applied Biosystems).

### Microsatellite data analysis

Allelic frequency, the number of alleles (*A*), observed heterozygosity (*H*_*o*_), expected heterozygosity (*H*_*e*_), and linkage equilibrium were calculated using Arlequin version 3.5.2.2 [31]. Since the population was small, deviations from Hardy-Weinberg equilibrium were also evaluated at each locus and population with the Markov chain Monte Carlo (MCMC) approximation of Fisher’s exact test using the “genepop” function in the package stats of R version 3.5.1 [32–34]. To test for equal variances between the *H*_*o*_ and *H*_*e*_ of all captive breeding programs, Bartlett’s test of homogeneity of variances was first conducted using the “bartlett.test” function in the package stats of R version 3.5.1 [34]. Welch’s t-test for unequal variance between samples was also conducted to test for significant differences between *H*_*o*_ and *H*_*e*,_ using the “t.test” function in the package stats of R version 3.5.1 [34,35]. Allelic richness (*AR*) was then calculated using FSTAT version 2.9.3 [36] and the mean number of effective alleles (*N*_*a*_) was obtained using GenAlEx version 6.5 [37]. We also compared *AR* among populations using the Kruskal-Wallis Test [38] with analysis by locus using the “kruskal.test” function in the package stats of R version 3.5.1 [34]. MicroChecker version 2.2.3 was used to determine null allelic markers [39]. Polymorphic information content (*PIC*) was estimated using the Excel Microsatellite Toolkit [40] and calculated for each locus and population. Shannon’s information index (*I*) and a fixation index (*F*) were also calculated for each locus of each population using GenAlEx version 6.5 [37]. Effective population size (*N*_*e*_) was estimated as the number of breeding individuals that contributed to the population using the linkage disequilibrium method in NeEstimator version 2.01 [41].

To consider the possibility of sibling or parent-offspring pairs in captive populations, we determined whether the Asian woolly-necked storks were more related than random unrelated individuals. Relatedness values (*r*) were calculated for all pairs of storks (comprising female-female, male-male, and male-female pairs) and mean pairwise *r* values, based on allelic frequencies of the population, were calculated at each captivity using GenAlEx version 6.5 [37]. The distribution of pairwise *r* values between all pairs from the sampled captivities was compared using a bootstrap version of the Kolmogorov-Smirnov test to provide relationships [42], using the “ks.test” function in the package stats of R version 3.5.1 [34]. Individual and overall inbreeding coefficients (*F*_IS_) with 95% confidence intervals were also calculated by LynchRt estimator [43] as implemented in COANCESTRY [44]. Examination of both *r* values and *F*_IS_ was conducted under the assumption that the averages did not differ significantly from random assortments of unrelated individuals. Parentage analysis and the probability that two individuals shared the same genotype were calculated using the COLONY program version 2.0.6.5 [45] and GIMLET version 1.3.3 [46], respectively. Mendelian inheritance was examined at every locus. Individuals sharing alleles from their putative parents at all loci were considered actual offspring of the couple. Those cases in which nestlings failed to match any of the two alleles of the putative parents at two or more loci were considered to be extra-pair paternity.

Pairwise genetic distances among populations were calculated based on the infinite allele model (IAM) using *F*_ST_ in Arlequin version 3.5.2.2. [31] with corrected *p* values, and the stepwise mutation model (SMM) using *R*_ST_ in FSTAT version 2.9.3. [36]. Considering possible influences of null alleles on genetic differentiation estimates, the FreeNA program [47] was also run, providing the pairwise *F*_ST_^ENA^ values with ENA correction for null alleles. To obtain a better understanding of group structure, an analysis of molecular variance (AMOVA) was performed using Arlequin 3.5.2.2 [27]. Unlike *F*_ST_, this algorithm identifies subgroup hierarchical structure and does not require a priori assumption of the Hardy-Weinberg equilibrium [27]. Nei’s genetic distances between groups were then examined using GenAlEx version 6.5 [37,48]. The state of heterozygosity excess and shift in allelic frequency distributions in genetically bottlenecked populations were tested using Bottleneck version 1.2.02 [49]. The Wilcoxon signed-rank test, with a two-phased model of mutation (TPM) and SMM, was used to obtain probabilities for excess levels of heterozygosity due to small sample sizes of loci and small sample size. The TPM was carried out with 95% single-step mutations and 5% multistep mutations, with variance among multiple steps set at 12 [49]. This test detects relatively short-term bottleneck events. To test for relatively long-term bottleneck events, the *M* ratio test [50] was performed using Arlequin version 3.5.2.2 [31]. The *M* ratio is the mean number of alleles in a population divided by the allelic size range and indicates reductions in both recent and historical population sizes. The model-based clustering method performance in Structure version 2.3.3 was used to determine population structure [51]. Run length was set to 100,000 Markov chain Monte Carlo replicates after a burn-in period of 100,000 generations, using correlated allelic frequencies under a straight admixture model. The number of clusters (*K*) was varied from 1 to 25, with 25 replicates for each value of *K*. The most likely number of clusters was determined by plotting the log probability of the data (ln Pr (*X*|*K*) [51] across the range of *K* values tested and selecting the *K* value at which ln Pr (*X*|*K*) stabilized. The Δ*K* method was also applied using Structure Harvester [52].

## Results

### Genetic diversity of the Asian woolly-necked stork in captive breeding programs

A total of 86 captive individuals (68 individuals from KKOZ, 16 from NRZ, and 2 from DSZ) were genotyped, and 68 alleles were detected among all loci, with a mean number of alleles per locus of 5.231 (Table 1, S3 Table). Null alleles were frequently found for six loci (Cc02, Cc04, Cc07, Cc10, Cbo109, and Cbo121); however, we treated all 13 markers listed in the table the same, including these. Allelic frequencies showed significant departures from Hardy-Weinberg expectations at four loci (Wsu13, Cc06, Cbo151, and Cbo108) of the stork population, with multiple lines of evidence for linkage disequilibrium (S3–S6 Tables). The ability to detect significant departures from Hardy-Weinberg equilibrium was limited due to the small sample sizes; however, consistent patterns of deviation from Hardy-Weinberg equilibrium or linkage equilibrium were not detected across sites. Consequently, genetic analyses were performed based on all microsatellite loci. All populations exhibited *F* values close to zero in NRZ and DSZ, but not in KKOZ. The *PIC* of all captive populations ranged from 0.000 to 0.710, and *I* ranged from 0.000 to 1.302 (Table 1, S3 Table). The *H*_o_ values ranged from 0.000 to 0.593 (0.417±0.148: mean ± SD) and the *H*_e_ values ranged from 0.000 to 0.751 (mean 0.524±0.200) (Table 1, S3 and S7 Tables). The *H*_o_ and *H*_e_ of KKOZ and NRZ populations were not statistically different (*H*_*o*_ of KKOZ and NRZ populations: t = -1.6824, df = 10.306, *p* = 0.1225 and *H*_*e*_ of KKOZ and NRZ populations: t = 0.5721, df = 18.648, *p* = 0.5741). Within each population, Welch’s t-test found that *H*_o_ was significantly different from *H*_e_ in the KKOZ population (*H*_o_ = 0.395±0.117, *H*_e_ = 0.520±0.213, df = 11, *p* < 0.05) but not in the NRZ population (*H*_o_ = 0.613±0.396, *H*_e_ = 0.466±0.230, df = 9, *p* = 0.1478). *AR* values of the KKOZ population were statistically higher than those of the NRZ population (D = 0.13663, *p* < 0.05). Standard genetic diversity indices are summarized in Table 1, S3 Table.

**Table 1:** Genetic diversity of 86 *Ciconia episcopus* individuals based on 13 microsatellite loci.

Locality	Locus	N	*A*	*AR*	*N*_*a*_	*I*	*H*_*o*_	*H*_*e*_	*M* ratio	*PIC*	*F*
Khao Kheow Open Zoo	Mean	68	5.154	4.054	2.400	0.963	0.395	0.520	0.292	0.443	0.163
	S.D.	0	0.839	2.114	0.330	0.159	0.117	0.213	0.217	0.243	0.076
Nakhon Ratchasima Zoo	Mean	16	2.385	2.385	1.887	0.578	0.613	0.466	0.254	0.294	-0.334
	S.D.	0	0.385	1.387	0.283	0.138	0.396	0.230	0.201	0.244	0.154
Dusit Zoo	Mean	2	1.538	N/A	1.451	0.326	0.833	0.639	0.278	0.176	-0.711
	S.D.	0	0.183	N/A	0.157	0.107	0.258	0.125	0.220	0.207	0.092
All populations	Mean	86	5.231	3.951	2.335	0.951	0.417	0.524	0.296	0.441	0.136
	S.D.	0	0.833	2.040	0.283	0.152	0.148	0.200	0.216	0.233	0.086

Sample size (N); number of alleles (*A*); Allelic richness (*AR*); number of effective alleles (*N*_*a*_); Shannon’s information index (*I*); observed heterozygosity (*H*_*o*_); expected heterozygosity (*H*_*e*_); M ratio test (*M* ratio); polymorphic information content values (*PIC*); fixation index (*F*); “N/A”: Not available.

### Relatedness and estimation of population size in captive populations

**Fig 1:**
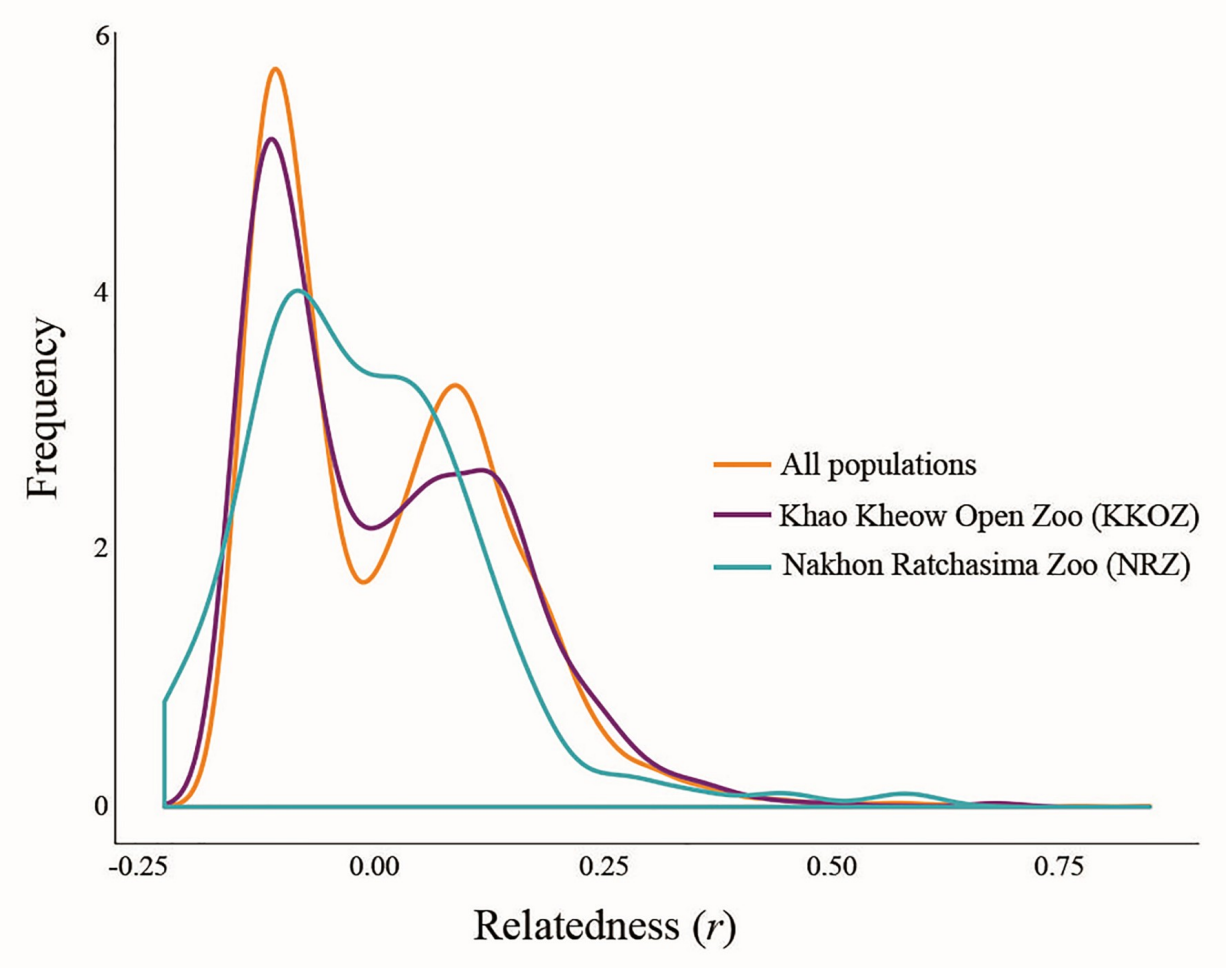
Observed distribution of pairwise relatedness (*r*) for 86 Asian woolly-necked storks (*Ciconia episcopus*) plotted against expected distributions.

A pairwise relatedness test was performed to determine the level of relatedness between individuals in all captive populations. The mean pairwise *r* value of 3,665 stork pairs among the 86 sampled storks was -0.007±0.115 (KKOZ population = -0.032±0.118 and NRZ population = -0.027±0.116). No stork pairs showed *r* < -0.25; there were 3,578 pairs with -0.25 < *r* < 0.25 (KKOZ population = 2,226 and NRZ population = 1,117), and 77 pairs with 0.25 < *r* (KKOZ population = 52 and NRZ population = 3) (Table 2, S8–S10 Tables), indicating that some proportions of the Asian woolly-necked stork pairs in each population were closely related (*r* > 0.25). Distributions of *r* values for the storks were skewed left, indicating pairwise *r* values lower than expected by chance from a null hypothesis of unrelated individuals. Relative to storks from all captivities, distributions of pairwise *r* values from KKOZ and NRZ populations were significantly different from each other and the mean of pairwise *r* values of all populations (KKOZ vs all populations: D = 0.047533, *p* < 0.01, NRZ vs all populations: D = 0.14995, *p* < 0.05, and KKOZ vs NRZ populations: D = 0.13663, *p* < 0.05) (Fig 1, Table 2). Mean *F*_IS_ was 0.107±0.210 (KKOZ population = 0.314±0.208 and NRZ population = -0.231±0.208), with individual *F*_IS_ ranging from -0.190 to 0.761 (Table 2, S11 Table).

**Table 2:** Inbreeding coefficients, relatedness, effective population size and ratio of effective population size and census population (*N*_*e*_/N) of *Ciconia episcopus* in Khao Kheow Open Zoo, Nakhon Ratchasima Zoo, and Dusit Zoo using NeEstimator version 2.01 [41], COANCESTRY [44] and GenAlEx version 6.5 [37], respectively. Detailed information for all *C*. *episcopus* individuals is presented in S1 Table.

Locality	N	*F*_IS_	Relatedness (*r*)	Estimated *N*_*e*_	95% CIs for *N*_*e*_	*N*_*e*_/N
Khao Kheow Open Zoo	68	0.134±0.208	-0.008 ± 0.118	1.7	1.5–1.9	0.025
Nakhon Ratchasima Zoo	16	-0.231±0.208	-0.027 ± 0.116	25.6	5.7–∞	1.6
Dusit Zoo	2	-0.654±0.093	N/A	N/A	N/A	N/A
Total	86	0.107±0.210	-0.007 ± 0.115	-	-	-

Inbreeding coefficients (*F*_IS_); effective population size (*N*_*e*_); “N/A”: Not available.

The *N*_*e*_ that genetically contributed to the population for KKOZ comprised 2 storks (95% CI: 1.5–1.9), and the *N*_*e*_ for the NRZ population comprised 26 storks (95% CI: 5.7–∞) (Table 2). Simultaneously, the parentage analysis from the captive stocks of all populations revealed that approximately one-third of all storks originated from three breeding pairs (29.07%) (KKOZ = 16.28%, NRZ = 10.47%, and DSZ = 2.33%), comprising at least 25 of the total number of storks. No genetic evidence of extra-pair paternity was found. All paternity assignments were assigned unequivocally. The combined probability of exclusion for the microsatellites used was estimated at 0.95. The likelihood of two individuals carrying an identical genotype was estimated at 3.11×10^−8^ (S12 Table).

### Population genetic structure and differentiation

After 110 permutations, estimates of *F*_ST_ showed significant differences between captive populations; however, estimates of *F*_ST_^ENA^ between captive populations were not different (S13 Table). AMOVA revealed that genetic variation was distributed mostly within each group (84.49% of variation), while only 15.51% was due to differences among groups (S14 Table). Nei’s genetic distances and *R*_ST_ showed that the most similar groups were the KKOZ and NRZ populations (S13 and S15 Tables). In Wilcoxon signed-rank tests for recent population bottlenecks, SMM and TPM were 0.997 and 0.455, respectively, in the KKOZ population (normal L-shaped mode shift) and 0.080 and 0.005, respectively, in the NRZ population (shifted mode). We could not analyze SMM and TPM in the DSZ population due to the small sample size (S16 Table). The *M* ratio across all populations averaged 0.292±0.217 for KKOZ and 0.254±0.201 for NRZ (Table 1, S3 and S7 Tables). These *M* ratio values were lower than the 0.68 threshold identified by Garza and Williamson [44], which indicates a historical reduction in population.

**Fig 2:**
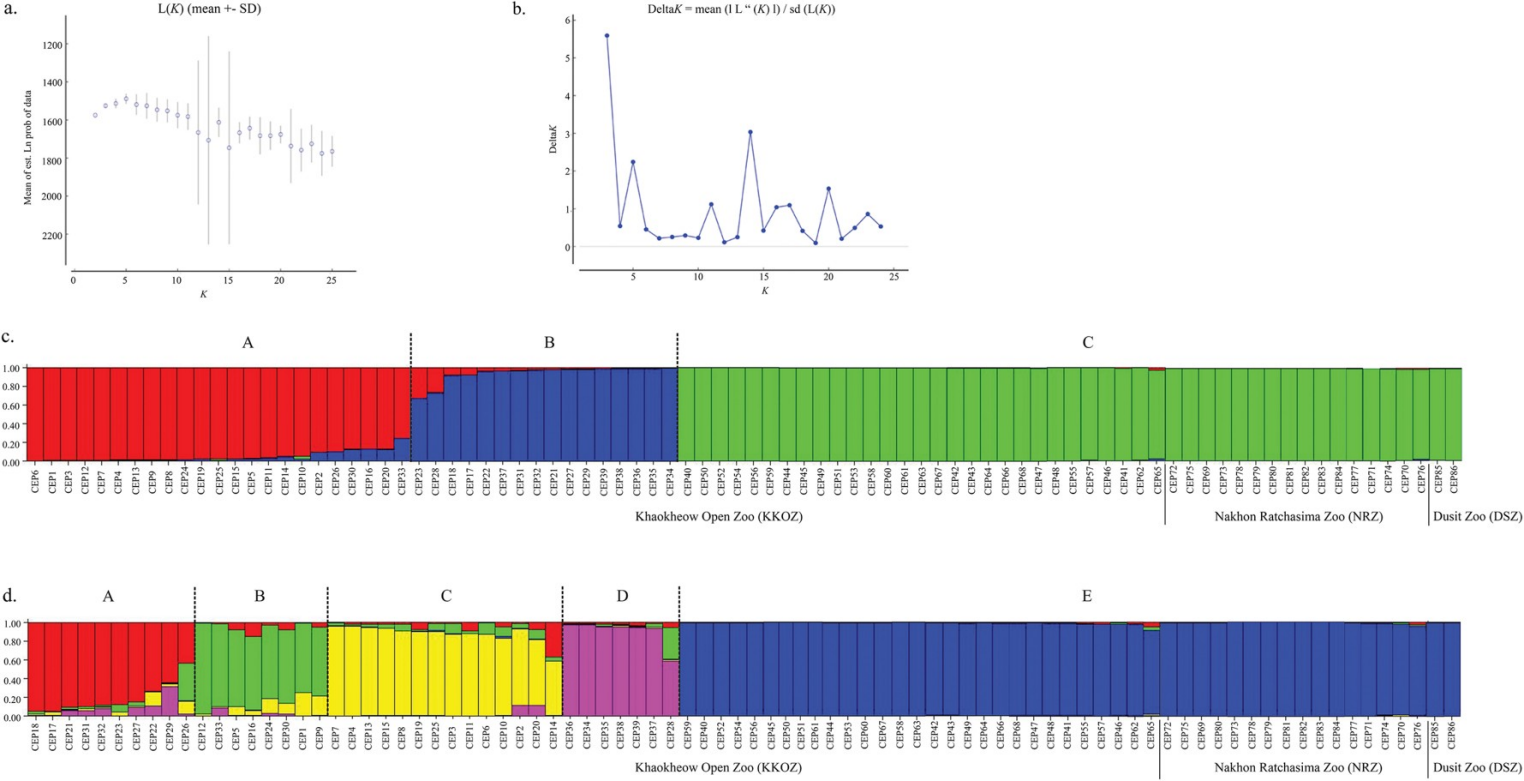
**Population structures of 86 Asian woolly-necked storks (*Ciconia episcopus*)** (a) Evanno’s Δ*K* graph. (b) Mean Ln P (*K*) graph, and Structure bar plots depicting model-based clustering results for inferred *K* = 3 (c) and *K* = 5 (d). Inferred genetic clusters are displayed as different colors. Each vertical bar on the x-axis represents an individual, and the y-axis presents the proportion of membership (posterior probability) in each genetic cluster. Recovered storks are superimposed on the plot, with black vertical lines indicating the boundaries. Detailed information for all stork individuals is presented in S1 Table.

Bayesian structural analysis revealed the highest posterior probability with one peak (*K* = 3) on the basis of Evanno’s Δ*K* with all storks grouped into three clusters (Fig 2a and 2c). This indicates that the KKOZ population was divided into three clusters but the NRZ and DSZ populations were grouped in the same cluster as a subdivision of the KKOZ population. By contrast, Bayesian structural analysis based on the mean ln P (*K*) revealed one peak (*K* = 5), which provided evidence for five clusters (Fig 2b and 2d). Four clusters of the KKOZ population were composed of only storks from the KKOZ population but only one cluster of the KKOZ population comprised storks from the NRZ and DSZ populations, as independent from the other four clusters.

## Discussion

In addition to an increased risk of extinction through demographic stochastic processes (inbreeding depression and loss of genetic diversity), a small population is detrimental [53,54]. Given the very small size of the Asian woolly-necked stork captive population, there is a need to establish intensive monitoring programs before, during, and beyond the initial reintroduction phase in order to ensure a long-term enduring population. The maintenance of genetic diversity and the understanding of demographic captive population structures are essential for improving the retention of genetic variation in small populations and contributing to adaptive management decisions [55].

### State of genetic diversity and demographic history in captive populations

The genetic diversity of all captive breeding programs consists of two informative forms: allelic variability and heterozygosity. Heterozygosity is sensitive to population growth rate, while allelic variability is sensitive to population size [56,57]. Here, *H*_*o*_ and *H*_*e*_ did not differ significantly between the KKOZ and NRZ populations; however, *H*_*e*_ was significantly higher than *H*_*o*_ in the KKOZ population. This suggests the possibility of inbreeding, resulting from a large number of generations in captivity and poor breeding success [14,58]. Although the *AR* values of the KKOZ population were higher than those of the NRZ population, both captive populations showed low *AR* values. This suggests that low *AR* values were derived from the loss of rare alleles, caused by the initial founding size of only a few storks during the 2000s [4]. However, sampling errors might have occurred as a consequence of the small sample size [59].

*F*_ST_ estimates revealed that while the captive KKOZ and NRZ populations differed significantly due to small population size [60], the *F*_ST_^ENA^ estimates were not different. AMOVA indicated that the largest proportion of variation occurred within populations (84.49%), and Nei’s pairwise genetic distance and *R*_ST_ comparison between the KKOZ and NRZ populations was also low when compared with the captive populations of other species [61]. *F* values of the KKOZ population were also positive. These results were consistent with Bayesian structural analysis, which exhibited several subdivisions in the KKOZ population. This suggests that genetic partition is a consequence of the possible mixed origin of captive populations, with founding individuals from various historically distinct lineages in the wild. Unfortunately, the original sources of the captive populations are unknown. Only one subdivision of the KKOZ population shared alleles with the NRZ and DSZ populations, suggesting genetic connectivity between the populations. In 2012, transfer occurred between the KKOZ and NRZ/DSZ populations in the absence of genetic diversity data, and managers rely solely on ethological, demographic, and logistic information for the implementation of short-term management strategies (Sudarath Baicharoen, personal communication). This suggests that shared genetic alleles among captive populations result from the movement of storks from KKOZ to other captivities. However, an additional bottleneck might have occurred during reintroduction, when the captive population was genetically sub-divided into several groups, including individuals, prior to release [16]. Therefore, careful examination of genetic variability within, and breeding plans for, species with low reproductive rates [62] or mating systems (where not all individuals contribute their genes to the next generation) can reduce the presence of inbreeding and genetic drift in captivity, allowing more adaptive management decisions.

### Breeding plan to contribute to *in situ* and *ex situ* management

Bottlenecks with low genetic diversity often occur when a small number of founders are taken from a declining wild population, resulting in poor breeding success [14]. This was also observed in our study where the *M* ratio signaled a historical reduction in all populations. A recent demographic bottleneck was not supported by the bottleneck test for the KKOZ population. Collectively, these results suggest that the KKOZ population underwent a recent expansion. An estimate of the ratio of *N*_*e*_ to consensus population (N) enables us to understand the population fitness including the risk arising from genetic factors [63]. The *N*_*e*_ and *N*_*e*_/N of the KKOZ population were extremely low and have generally remained low, relative to the captive populations of other species [64,65]. This points to the loss of genetic variation through genetic drift, wherein a small founding KKOZ population was likely composed of related individuals. Alternatively, multiple generations in the KKOZ population are critical to reducing population size and increased inbreeding, as found in other animal captive populations [14,58]. By contrast, the occurrence of a recent demographic bottleneck was supported by the bottleneck test for the NRZ population. The *N*_*e*_ of the NRZ population was low, but the *N*_*e*_/N value was greater than one. This suggests that genetic drift, with a possible recent and mixed origin of the initial population, occurred in NRZ. The occurrence of monogynous behaviors and lack of extra-pair fertilizations were likely to cause a reduction in *N*_*e*_ [66,67]. Populations should be composed of young males and/or skewed toward females that have more equitable breeding opportunities [68,69]. An increase in *N*_*e*_ and a management strategy including long-term gene flow between captive populations are required to mitigate genetic drift, especially when they are expected to function as sources of genetic variation for an *in situ* program.

**Fig 3:**
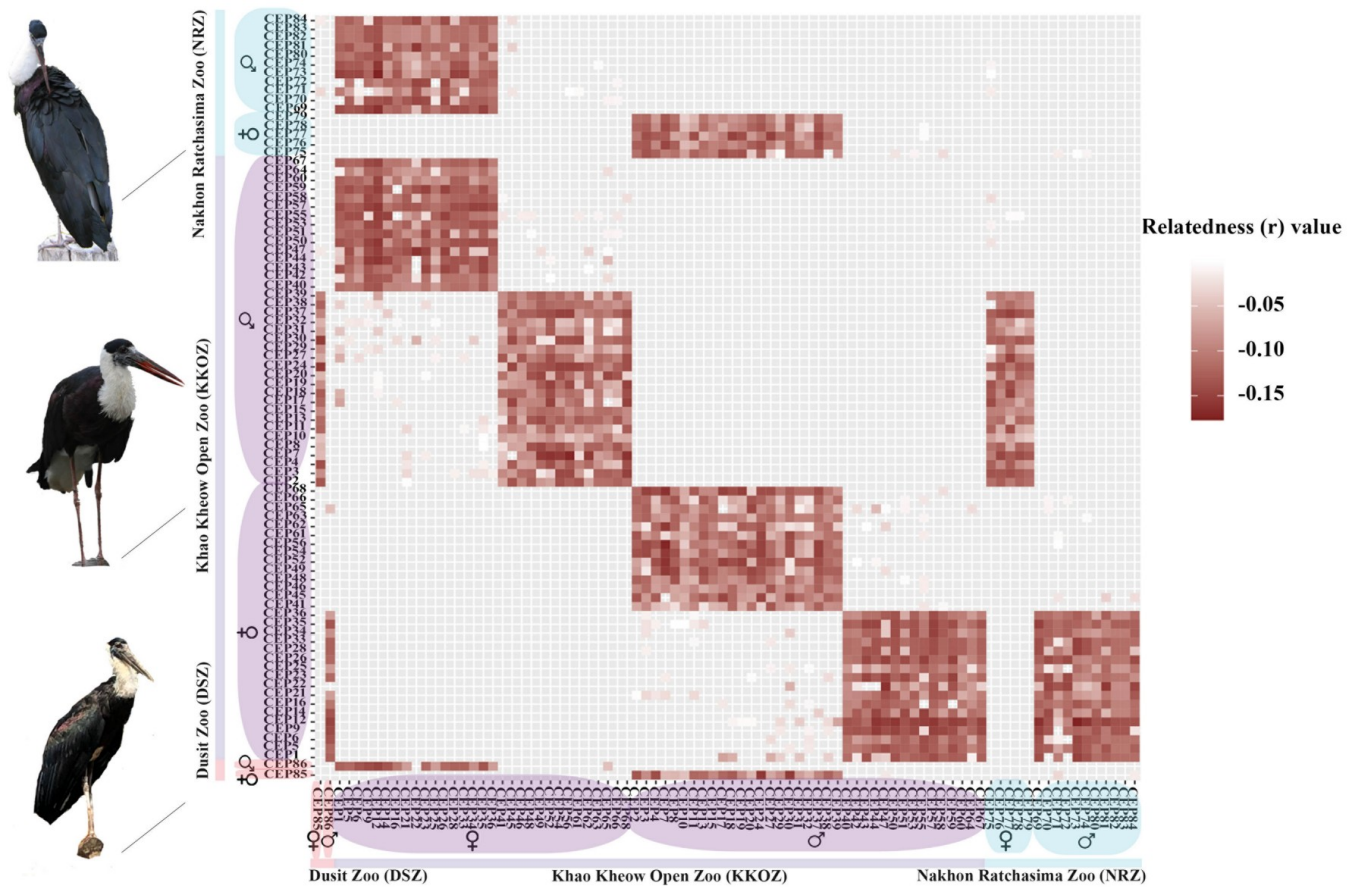
Heat map of pairwise relatedness (*r*) values illustrating Asian woolly-necked stork (*Ciconia episcopus*) captive populations from Khao Kheow Open Zoo (KKOZ), Nakhon Ratchasima Zoo (NRZ), and Dusit Zoo (DSZ). Values closer to zero (white) signify high relatedness whereas values closer to large minuses (red) are low relatedness across 13 microsatellite loci. Colored regions to the left and at the bottom correspond to source captive populations. The matrix is clustered by phenotypic males and females, as indicated by symbols left and bottom.

Before moving forward with the captive breeding program and reintroducing storks, we should take one step backward and consider *N*_*e*_ in relation to inbreeding states in KKOZ and NRZ captive populations. The positive *F*_IS_ value in the KKOZ population is indicative of inbreeding; on the other hand, a negative *F*_IS_ in the NRZ population indicates outbreeding, consistent with the historical transfer. Distribution of pairwise *r* values in the KKOZ population was also significantly different from the NRZ population. In New Zealand, the Takahe (*Porphyrio hochstetteri*) are managed to maintain genetic variation and reduce inbreeding by removing individuals with high *r* values from the population and replacing them with unrelated individuals [70]. In this study, the mean *r* value was near zero in the NRZ population, indicating the possibility of unrelated individuals being introduced, rather than related storks, as in KKOZ. The minimization of relatedness within the source population is necessary to promote successful management action [71]. By prioritizing individuals for breeding pairs with low relatedness, population-level inbreeding and the loss of genetic diversity can be mitigated by equalizing representation of individual genetic material within a population (Fig 3) [72]. In future transfers, a low relatedness strategy could be implemented to prioritize individuals for transfer between the KKOZ and NRZ populations. Cryopreservation of sperm and oocytes has also been considered for the conservation of the Asian woolly-necked stork, in order to preserve the genetic diversity of their stocks under the limitation of effective population size [73]. However, while we note that cryopreservation is not always possible when taking action on conservation, we suggest that genetic diversity should be fully assessed prior to any recommendations for *ex situ* management. These could include the frequent introduction of new individuals into gene pools, breeding protocols to maintain high levels of genetic diversity in captive populations, and selection of release groups. Ideally, the state of genetic diversity for a captive population is determined by the proportion of high heterozygosity under a specific time. This also depends on the rate of species reproduction and generation length [74]. Integral management and conservation strategies such as examination/monitoring of physical health and the likelihood of behavioral anomalies are also required for future adaptations to environmental change, both in captivity and in the wild [54,75,76]. The most important step in future studies will be consideration of habitat. The search for suitable habitats for the introduction of Asian woolly-necked storks is an additional management action required for Thailand, to ensure the maintenance of natural diversity and structure as part of the species’ overall conservation plan. We recommend collaboration with environmental authorities to design and improve habitats, to allocate financial resources, and also to provide populations with protection from harvesting and illegal kills. The release site should be within the historic range of the Asian woolly-necked stork [4,5]. Our findings could help to streamline conservation efforts for this species in Thailand.

Directed efforts (e.g. breeding practice, transfer, and reintroduction) are underway in Thailand to protect and restore populations of Asian woolly-necked stork through *in situ* and *ex situ* management strategies. It is clear that the assessment of genetic diversity is an excellent means of maximizing reproductive success and promoting genetic variation in captive-bred individuals for subsequent release into the wild or to supplement captive stocks [11,77]. The maintenance of long-term enduring populations requires the consideration of accurate genetic breeding plans. Our findings are a first step in the establishment of captive breeding and reintroduction programs in the National Action Plan. Our results should be considered with caution before being implemented in strict management or conservation strategies, to avoid genetic drift and to ensure that a high proportion of the source variation is settled to minimize loss of genetic diversity, given that our sample sizes are very small. Transfers between captive populations are desirable in the search for greater genetic diversity; however, genetic monitoring is costly and time-consuming and beyond the budget of several conservation initiatives. Experiences gained from other programs of captive breeding and reintroduction could be of great value in terms of developing our understanding of these matters.

## Supporting information

S1 Table Summary of *Ciconia episcopus* specimens. (DOCX)

S2 Table Microsatellite primers and sequences. (DOCX)

S3 Table Genetic diversity of 86 *Ciconia episcopus* individuals based on 13 microsatellite loci. Detailed information for all *C*. *episcopus* individuals is presented in S1 Table.
(DOCX)

S4 Table Pairwise differentiation of linkage disequilibrium of *Ciconia episcopus* individuals in Khao Kheow Open Zoo based on 13 microsatellite loci. The number indicates *p* values, with 110 permutations.
(DOCX)

S5 Table Pairwise differentiation of linkage disequilibrium of *Ciconia episcopus* individuals in Nakhon Ratchasima Zoo based on 13 microsatellite loci. The number indicates *p* values, with 110 permutations.
(DOCX)

S6 Table Pairwise differentiation of linkage disequilibrium of *Ciconia episcopus* individuals in Dusit Zoo based on 13 microsatellite loci. The number indicates *p* values, with 110 permutations.
(DOCX)

S7 Table Observed and expected heterozygosity of *Ciconia episcopus* based on 13 microsatellite loci in each captive breeding. Detailed information for all *C*. *episcopus* individuals is presented in S1 Table.
(DOCX)

S8 Table Pairwise genetic relatedness (*r*) for all 86 *Ciconia episcopus* individuals. Detailed information for all *C*. *episcopus* individuals is presented in S1 Table.
(DOCX)

S9 Table Pairwise genetic relatedness (*r*) for all 68 *Ciconia episcopus* individuals in Khao Kheow Open Zoo. Detailed information for all *C*. *episcopus* individuals is presented in S1 Table.
(DOCX)

S10 Table Pairwise genetic relatedness (*r*) for all 16 *Ciconia episcopus* individuals in Nakhon Ratchasima Zoo. Detailed information for all *C*. *episcopus* individuals is presented in S1 Table.
(DOCX)

S11 Table Pairwise inbreeding coefficients (*F*_IS_) for all 86 *Ciconia episcopus* individuals. Detailed information for all *C*. *episcopus* individuals is presented in S1 Table.
(DOCX)

S12 Table Probability of identity using GIMLET version 1.3.3 [46] of *Ciconia episcopus* individuals based on 13 microsatellite loci. Detailed information for all *C*. *episcopus* individuals is presented in S1 Table.
(DOCX)

S13 Table Pairwise genetic differentiation (*F*_*ST*_), pairwise *F*STENA values with ENA correction for null alleles and *R*_ST_ values using FSTAT version 2.9.3 [36] and of *Ciconia episcopus* between captive breeding based on 13 microsatellite loci. The number indicates *p* values, with 110 permutations. Detailed information for all *C*. *episcopus* individuals is presented in S1 Table.
(DOCX)

S14 Table Analysis of molecular variance (AMOVA) results of *Ciconia episcopus* based on 13 microsatellite loci using Arlequin version 3.5.2.2 [31]. Detailed information for all *C*. *episcopus* individuals is presented in S1 Table.
(DOCX)

S15 Table Pairwise population Nei’s genetic distance (*D*) values using GenAlEx version 6.5 [37] of 86 *Ciconia episcopus* individuals in each zoo based on 13 microsatellite loci. (DOCX)

S16 Table Genetic bottlenecks of *Ciconia episcopus* individuals in the three zoos using BOTTLENECK version 1.2.02 [49] and calculation of *M* ratio using Arlequin version 3.5.2.2 [27] for all populations. Detailed information for all *C*. *episcopus* individuals is presented in S1 Table.
(DOCX)
