# Supplementary material for: Take one step backward to move forward: Assessment of genetic diversity and population structure of captive Asian woolly-necked storks (Ciconia episcopus)
Source: PLoS One. 2019 Oct 10;14(10):e0223726. doi: 10.1371/journal.pone.0223726 (PMC6786576; doi:10.1371/journal.pone.0223726)
Supplement: S1 Table — (DOCX) [file pone.0223726.s001.docx]

**S1 Table.** Summary of *Ciconia episcopus* specimens.

| Number | Locality | Locality code | Specimen no. | Code |
| --- | --- | --- | --- | --- |
| # 1 | Khao Kheow Open Zoo | KKOZ | 68 | CEP1–68 |
| # 2 | Nakhon Ratchasima Zoo | NRZ | 16 | CEP69–84 |
| # 3 | Dusit Zoo | DSZ | 2 | CEP85–86 |
