# Supplementary material for: Take one step backward to move forward: Assessment of genetic diversity and population structure of captive Asian woolly-necked storks (Ciconia episcopus)
Source: PLoS One. 2019 Oct 10;14(10):e0223726. doi: 10.1371/journal.pone.0223726 (PMC6786576; doi:10.1371/journal.pone.0223726)
Supplement: S2 Table — (DOCX) [file pone.0223726.s002.docx]

**S2 Table.** Microsatellite primers and sequences.

| Primer | Primer sequence 5′ to 3′ | Reference |
| --- | --- | --- |
| Wsu13 | AGGGCTCATCAATAGTGT  GTTTGCCCACTGTGTCAACT | [24] |
| Ah211 | GCTCATCAGGAGTTGAATCTGGC  TCTGTCATTCAGCAATGGACC | [25] |
| Cc02 | CGCTCGCTGTCTTTATCTCC  CTGCTGCATGCCAGTTGAT | [25] |
| Cc04 | VICGCTGAAATGTCTGTCCCTGA  AATTCCCCTATTGCGTCACA | [23] |
| Cc06 | CTCGCTGTCTCCTCTGCTCT  GAACAGCAATATCGCATCTACA | [25] |
| Cc07 | GCATGAAAATGCATAGAGCAGA  CCACCGTTATGATCCTTTGG | [23] |
| Cc10 | TGTGACAGATGCAAAGCTCC  GTGTTTACTAGTTGGCTGTTCC | [26] |
| Cc37 | CCTGCCTGACAAGAGAATGC  GCAAGTGTATCAGTCCAAATGG | [26] |
| Cc42 | GCAGGAAAGGAGGAAAGGTG  GCATCACAGTATGCAAACGC | [26] |
| Cbo108 | CCCAGGTCACAAATTATACG  GAGCCTCACAAAGTTCCCTG | [24] |
| Cbo109 | GTGGTGTAGTCCAGTTTATG  ATAACACATGAATGACCTGG | [24] |
| Cbo121 | CCACAATGGCAATTTTTCAC  GTTCTCCCAGAGGCTTGCTC | [24] |
| Cbo151 | AATCTGGTCTTGGTCCTTTC  GGTTTTACCCTCTGACACTG | [24] |
