# Supplementary material for: Take one step backward to move forward: Assessment of genetic diversity and population structure of captive Asian woolly-necked storks (Ciconia episcopus)
Source: PLoS One. 2019 Oct 10;14(10):e0223726. doi: 10.1371/journal.pone.0223726 (PMC6786576; doi:10.1371/journal.pone.0223726)
Supplement: S3 Table — Detailed information for all C. episcopus individuals is presented in S1 Table. (DOCX) [file pone.0223726.s003.docx]

**S3 Table.** Genetic diversity of 86 *Ciconia episcopus* individuals based on 13 microsatellite loci. Detailed information for all *C. episcopus* individuals is presented in S1 Table.

| Locality | Locus | N | *A* | *AR* | *N_a_* | *I* | *H_o_* | *H_e_* | *M* ratio | *PIC* | *F* | HW-test |
| --- | --- | --- | --- | --- | --- | --- | --- | --- | --- | --- | --- | --- |
| Khao Kheow Open Zoo | Wsu13 | 68 | 3 | 2.414 | 1.380 | 0.492 | 0.324 | 0.278 | 0.024 | 0.245 | -0.174 | 0.523 |
|  | Cc10 | 68 | 6 | 5.788 | 4.130 | 1.588 | 0.412 | 0.764 | 0.273 | 0.726 | 0.457 | 0.000 |
|  | Ah211 | 68 | 4 | 3.890 | 2.783 | 1.168 | 0.485 | 0.645 | 0.190 | 0.586 | 0.243 | 0.000 |
|  | Cc02 | 68 | 2 | 1.999 | 1.391 | 0.454 | 0.162 | 0.283 | 0.667 | 0.242 | 0.424 | 0.002 |
|  | Cc06 | 68 | 3 | 2.409 | 1.323 | 0.451 | 0.279 | 0.246 | 0.600 | 0.221 | -0.145 | 0.682 |
|  | Cc42 | 68 | 3 | 2.416 | 1.558 | 0.591 | 0.426 | 0.361 | 0.091 | 0.304 | -0.190 | 0.001 |
|  | Cbo121 | 68 | 8 | 6.497 | 2.748 | 1.430 | 0.426 | 0.641 | 0.471 | 0.612 | 0.330 | 0.000 |
|  | Cc07 | 68 | 9 | 6.872 | 4.293 | 1.699 | 0.559 | 0.773 | 0.148 | 0.735 | 0.271 | 0.000 |
|  | Cbo109 | 68 | 8 | 6.927 | 3.967 | 1.680 | 0.559 | 0.753 | 0.533 | 0.723 | 0.253 | 0.000 |
|  | Cc04 | 68 | 11 | 6.287 | 3.216 | 1.504 | 0.294 | 0.694 | 0.129 | 0.651 | 0.573 | 0.000 |
|  | Cbo151 | 68 | 4 | 3.174 | 1.962 | 0.851 | 0.441 | 0.494 | 0.200 | 0.427 | 0.100 | 0.076 |
|  | Cbo108 | 68 | 5 | 3.024 | 1.453 | 0.604 | 0.368 | 0.314 | 0.172 | 0.282 | -0.180 | 0.650 |
|  | Cc37 | 68 | 1 | 1.000 | 1.000 | 0.000 | 0.000 | 0.000 | 0.000 | 0.000 | N/A | N/A |
|  | Mean | 68 | 5.154 | 4.054 | 2.400 | 0.963 | 0.395 | 0.520 | 0.292 | 0.443 | 0.163 | - |
|  | S.D. | 0 | 0.839 | 2.114 | 0.330 | 0.159 | 0.117 | 0.213 | 0.217 | 0.243 | 0.076 | - |
| Nakhon Ratchasima Zoo | Wsu13 | 16 | 1 | 1 | 1.000 | 0.000 | 0.000 | 0.000 | 0.000 | 0.000 | N/A | N/A |
|  | Cc10 | 16 | 2 | 2 | 1.064 | 0.139 | 0.063 | 0.063 | 0.143 | 0.059 | -0.032 | N/A |
|  | Ah211 | 16 | 2 | 2 | 2.000 | 0.693 | 1.000 | 0.516 | 0.095 | 0.375 | -1.000 | 0.000 |
|  | Cc02 | 16 | 2 | 2 | 1.205 | 0.311 | 0.188 | 0.175 | 0.667 | 0.155 | -0.103 | 1.000 |
|  | Cc06 | 16 | 2 | 2 | 1.358 | 0.433 | 0.313 | 0.272 | 0.400 | 0.229 | -0.185 | 1.000 |
|  | Cc42 | 16 | 2 | 2 | 2.000 | 0.693 | 1.000 | 0.516 | 0.061 | 0.375 | -1.000 | 0.000 |
|  | Cbo121 | 16 | 6 | 6 | 4.571 | 1.619 | 1.000 | 0.806 | 0.400 | 0.748 | -0.280 | 0.462 |
|  | Cc07 | 16 | 4 | 4 | 2.994 | 1.197 | 0.688 | 0.688 | 0.129 | 0.602 | -0.032 | 0.016 |
|  | Cbo109 | 16 | 1 | 1 | 1.000 | 0.000 | 0.000 | 0.000 | 0.000 | 0.000 | N/A | N/A |
|  | Cc04 | 16 | 3 | 3 | 2.327 | 0.947 | 0.125 | 0.589 | 0.075 | 0.496 | 0.781 | 0.000 |
|  | Cbo151 | 16 | 3 | 3 | 2.024 | 0.786 | 0.813 | 0.522 | 0.167 | 0.406 | -0.606 | 0.031 |
|  | Cbo108 | 16 | 2 | 2 | 1.992 | 0.691 | 0.938 | 0.514 | 0.400 | 0.374 | -0.882 | 0.001 |
|  | Cc37 | 16 | 1 | 1 | 1.000 | 0.000 | 0.000 | 0.000 | 0.000 | 0.000 | N/A | N/A |
|  | Mean | 16 | 2.385 | 2.385 | 1.887 | 0.578 | 0.613 | 0.466 | 0.254 | 0.294 | -0.334 | - |
|  | S.D. | 0 | 0.385 | 1.387 | 0.283 | 0.138 | 0.396 | 0.230 | 0.201 | 0.244 | 0.154 | - |
| Dusit Zoo | Wsu13 | 2 | 1 | N/A | 1.000 | 0.000 | 0.000 | 0.000 | 0.000 | 0.000 | N/A | N/A |
|  | Cc10 | 2 | 1 | N/A | 1.000 | 0.000 | 0.000 | 0.000 | 0.000 | 0.000 | N/A | N/A |
|  | Ah211 | 2 | 2 | N/A | 2.000 | 0.693 | 1.000 | 0.667 | 0.095 | 0.375 | -1.000 | 1.000 |
|  | Cc02 | 2 | 1 | N/A | 1.000 | 0.000 | 0.000 | 0.000 | 0.000 | 0.000 | N/A | N/A |
|  | Cc06 | 2 | 2 | N/A | 1.600 | 0.562 | 0.500 | 0.500 | 0.400 | 0.305 | -0.333 | N/A |
|  | Cc42 | 2 | 2 | N/A | 2.000 | 0.693 | 1.000 | 0.667 | 0.061 | 0.375 | -1.000 | 1.000 |
|  | Cbo121 | 2 | 3 | N/A | 2.667 | 1.040 | 1.000 | 0.833 | 0.600 | 0.555 | -0.600 | 1.000 |
|  | Cc07 | 2 | 1 | N/A | 1.000 | 0.000 | 0.000 | 0.000 | 0.000 | 0.000 | N/A | N/A |
|  | Cbo109 | 2 | 1 | N/A | 1.000 | 0.000 | 0.000 | 0.000 | 0.000 | 0.000 | N/A | N/A |
|  | Cc04 | 2 | 1 | N/A | 1.000 | 0.000 | 0.000 | 0.000 | 0.000 | 0.000 | N/A | N/A |
|  | Cbo151 | 2 | 2 | N/A | 2.000 | 0.693 | 1.000 | 0.667 | 0.111 | 0.375 | -1.000 | 1.000 |
|  | Cbo108 | 2 | 2 | N/A | 1.600 | 0.562 | 0.500 | 0.500 | 0.400 | 0.305 | -0.333 | N/A |
|  | Cc37 | 2 | 1 | N/A | 1.000 | 0.000 | 0.000 | 0.000 | 0.000 | 0.000 | N/A | N/A |
|  | Mean | 2 | 1.538 | N/A | 1.451 | 0.326 | 0.833 | 0.639 | 0.278 | 0.176 | -0.711 | - |
|  | S.D. | 0 | 0.183 | N/A | 0.157 | 0.107 | 0.258 | 0.125 | 0.220 | 0.207 | 0.092 | - |
| All populations | Wsu13 | 86 | 3.000 | 2.335 | 1.292 | 0.421 | 0.256 | 0.227 | 0.024 | 0.205 | -0.133 | 0.688 |
|  | Cc10 | 86 | 6.000 | 5.687 | 3.115 | 1.431 | 0.337 | 0.683 | 0.273 | 0.648 | 0.503 | 0.000 |
|  | Ah211 | 86 | 4.000 | 3.823 | 3.153 | 1.218 | 0.593 | 0.687 | 0.190 | 0.621 | 0.132 | 0.000 |
|  | Cc02 | 86 | 2.000 | 1.998 | 1.404 | 0.463 | 0.163 | 0.290 | 0.667 | 0.247 | 0.435 | 0.000 |
|  | Cc06 | 86 | 3.000 | 2.339 | 1.336 | 0.455 | 0.291 | 0.253 | 0.600 | 0.225 | -0.156 | 0.511 |
|  | Cc42 | 86 | 3.000 | 2.346 | 1.706 | 0.646 | 0.547 | 0.416 | 0.091 | 0.337 | -0.321 | 0.000 |
|  | Cbo121 | 86 | 8.000 | 6.622 | 3.783 | 1.635 | 0.547 | 0.740 | 0.471 | 0.709 | 0.257 | 0.000 |
|  | Cc07 | 86 | 9.000 | 6.582 | 3.947 | 1.626 | 0.570 | 0.751 | 0.148 | 0.710 | 0.237 | 0.000 |
|  | Cbo109 | 86 | 8.000 | 6.614 | 2.878 | 1.472 | 0.442 | 0.656 | 0.533 | 0.629 | 0.323 | 0.000 |
|  | Cc04 | 86 | 11.000 | 5.893 | 3.027 | 1.439 | 0.256 | 0.674 | 0.129 | 0.631 | 0.618 | 0.000 |
|  | Cbo151 | 86 | 5.000 | 3.268 | 2.105 | 0.895 | 0.523 | 0.528 | 0.250 | 0.444 | 0.003 | 0.111 |
|  | Cbo108 | 86 | 5.000 | 2.852 | 1.602 | 0.665 | 0.477 | 0.378 | 0.172 | 0.324 | -0.269 | 0.088 |
|  | Cc37 | 86 | 1.000 | 1.000 | 1.000 | 0.000 | 0.000 | 0.000 | 0.000 | 0.000 | N/A | N/A |
|  | Mean | 86 | 5.231 | 3.951 | 2.335 | 0.951 | 0.417 | 0.524 | 0.296 | 0.441 | 0.136 | - |
|  | S.D. | 0 | 0.833 | 2.040 | 0.283 | 0.152 | 0.148 | 0.200 | 0.216 | 0.233 | 0.086 | - |

Sample size (N); number of alleles (*A*); Allelic richness (*AR*); number of effective alleles (*N_a_*); Shannon’s information index (*I*); observed heterozygosity (*H_o_*); expected heterozygosity (*H_e_*); M ratio test (*M* ratio); polymorphic information content values (*PIC*); fixation index (*F*); HW test, *p* value of the test for Hardy-Weinberg equilibriu based on the Markov chain Monte Carlo (MCMC) approximation of Fisher’s exact test; “N/A”: Not available.
