# Supplementary material for: Take one step backward to move forward: Assessment of genetic diversity and population structure of captive Asian woolly-necked storks (Ciconia episcopus)
Source: PLoS One. 2019 Oct 10;14(10):e0223726. doi: 10.1371/journal.pone.0223726 (PMC6786576; doi:10.1371/journal.pone.0223726)
Supplement: S4 Table — The number indicates p values, with 110 permutations. (DOCX) [file pone.0223726.s004.docx]

**S4 Table.** Pairwise differentiation of linkage disequilibrium of *Ciconia episcopus* individuals in Khao Kheow Open Zoo based on 13 microsatellite loci. The number indicates *p* values, with 110 permutations.

| Locus | Wsu13 | Cc10 | Ah211 | Cc02 | Cc06 | Cc42 | Cbo121 | Cc07 | Cbo109 | Cc04 | | Cbo151 | Cbo108 | Cc37 |
| --- | --- | --- | --- | --- | --- | --- | --- | --- | --- | --- | --- | --- | --- | --- |
| Wsu13 | 0.000 |  |  |  |  |  |  |  |  |  |  | |  |  |
| Cc10 | 0.000 | 0.000 |  |  |  |  |  |  |  |  |  | |  |  |
| Ah211 | 0.000 | 0.000 | 0.000 |  |  |  |  |  |  |  |  | |  |  |
| Cc02 | 0.911 | 0.336 | 0.441 | 0.000 |  |  |  |  |  |  |  | |  |  |
| Cc06 | 0.567 | 0.118 | 0.534 | 0.024 | 0.000 |  |  |  |  |  |  | |  |  |
| Cc42 | 0.001 | 0.000 | 0.000 | 0.401 | 0.295 | 0.000 |  |  |  |  |  | |  |  |
| Cbo121 | 0.000 | 0.000 | 0.000 | 0.675 | 0.023 | 0.000 | 0.000 |  |  |  |  | |  |  |
| Cc07 | 0.220 | 0.021 | 0.004 | 0.855 | 0.617 | 0.009 | 0.000 | 0.000 |  |  |  | |  |  |
| Cbo109 | 0.000 | 0.000 | 0.000 | 0.530 | 0.549 | 0.000 | 0.000 | 0.000 | 0.000 |  |  | |  |  |
| Cc04 | 0.004 | 0.000 | 0.000 | 0.116 | 0.272 | 0.005 | 0.000 | 0.043 | 0.000 | 0.000 |  | |  |  |
| Cbo151 | 0.003 | 0.000 | 0.000 | 0.396 | 0.040 | 0.000 | 0.000 | 0.017 | 0.000 | 0.000 | 0.000 | |  |  |
| Cbo108 | 0.029 | 0.000 | 0.000 | 0.184 | 0.833 | 0.000 | 0.000 | 0.020 | 0.000 | 0.054 | 0.000 | | 0.000 |  |
| Cc37 | 1.000 | 1.000 | 1.000 | 1.000 | 1.000 | 1.000 | 1.000 | 1.000 | 1.000 | 1.000 | 1.000 | | 1.000 | 0.000 |
