# Supplementary material for: Take one step backward to move forward: Assessment of genetic diversity and population structure of captive Asian woolly-necked storks (Ciconia episcopus)
Source: PLoS One. 2019 Oct 10;14(10):e0223726. doi: 10.1371/journal.pone.0223726 (PMC6786576; doi:10.1371/journal.pone.0223726)
Supplement: S5 Table — The number indicates p values, with 110 permutations. (DOCX) [file pone.0223726.s005.docx]

**S5 Table.** Pairwise differentiation of linkage disequilibrium of *Ciconia episcopus* individuals in Nakhon Ratchasima Zoo based on 13 microsatellite loci. The number indicates *p* values, with 110 permutations.

| Locus | Wsu13 | Cc10 | Ah211 | Cc02 | Cc06 | Cc42 | Cbo121 | Cc07 | Cbo109 | Cc04 | Cbo151 | Cbo108 | Cc37 |
| --- | --- | --- | --- | --- | --- | --- | --- | --- | --- | --- | --- | --- | --- |
| Wsu13 | 0.000 |  |  |  |  |  |  |  |  |  |  |  |  |
| Cc10 | 1.000 | 0.000 |  |  |  |  |  |  |  |  |  |  |  |
| Ah211 | 1.000 | 0.718 | 0.000 |  |  |  |  |  |  |  |  |  |  |
| Cc02 | 1.000 | 1.000 | 0.723 | 0.000 |  |  |  |  |  |  |  |  |  |
| Cc06 | 1.000 | 1.000 | 0.454 | 0.197 | 0.000 |  |  |  |  |  |  |  |  |
| Cc42 | 1.000 | 0.629 | 0.000 | 0.447 | 0.276 | 0.000 |  |  |  |  |  |  |  |
| Cbo121 | 1.000 | 0.214 | 0.170 | 0.103 | 0.320 | 0.174 | 0.000 |  |  |  |  |  |  |
| Cc07 | 1.000 | 0.281 | 0.216 | 0.702 | 0.595 | 0.227 | 0.000 | 0.000 |  |  |  |  |  |
| Cbo109 | 1.000 | 1.000 | 1.000 | 1.000 | 1.000 | 1.000 | 1.000 | 1.000 | 0.000 |  |  |  |  |
| Cc04 | 1.000 | 0.432 | 1.000 | 0.285 | 0.902 | 1.000 | 0.659 | 0.025 | 1.000 | 0.000 |  |  |  |
| Cbo151 | 1.000 | 0.522 | 0.013 | 0.574 | 0.343 | 0.012 | 0.368 | 0.727 | 1.000 | 0.624 | 0.000 |  |  |
| Cbo108 | 1.000 | 0.733 | 0.001 | 0.281 | 0.375 | 0.001 | 0.013 | 0.427 | 1.000 | 0.274 | 0.028 | 0.000 |  |
| Cc37 | 1.000 | 1.000 | 1.000 | 1.000 | 1.000 | 1.000 | 1.000 | 1.000 | 1.000 | 1.000 | 1.000 | 1.000 | 0.000 |
