# Supplementary material for: Take one step backward to move forward: Assessment of genetic diversity and population structure of captive Asian woolly-necked storks (Ciconia episcopus)
Source: PLoS One. 2019 Oct 10;14(10):e0223726. doi: 10.1371/journal.pone.0223726 (PMC6786576; doi:10.1371/journal.pone.0223726)
Supplement: S6 Table — The number indicates p values, with 110 permutations. (DOCX) [file pone.0223726.s006.docx]

**S6 Table.** Pairwise differentiation of linkage disequilibrium of *Ciconia episcopus* individuals in Dusit Zoo based on 13 microsatellite loci. The number indicates *p* values, with 110 permutations.

| Locus | Wsu13 | Cc10 | Ah211 | Cc02 | Cc06 | Cc42 | Cbo121 | Cc07 | Cbo109 | Cc04 | Cbo151 | Cbo108 | Cc37 |
| --- | --- | --- | --- | --- | --- | --- | --- | --- | --- | --- | --- | --- | --- |
| Wsu13 | 0.000 |  |  |  |  |  |  |  |  |  |  |  |  |
| Cc10 | 1.000 | 0.000 |  |  |  |  |  |  |  |  |  |  |  |
| Ah211 | 1.000 | 1.000 | 0.000 |  |  |  |  |  |  |  |  |  |  |
| Cc02 | 1.000 | 1.000 | 1.000 | 0.000 |  |  |  |  |  |  |  |  |  |
| Cc06 | 1.000 | 1.000 | 0.958 | 1.000 | 0.000 |  |  |  |  |  |  |  |  |
| Cc42 | 1.000 | 1.000 | 0.437 | 1.000 | 0.780 | 0.000 |  |  |  |  |  |  |  |
| Cbo121 | 1.000 | 1.000 | 0.785 | 1.000 | 0.672 | 0.917 | 0.000 |  |  |  |  |  |  |
| Cc07 | 1.000 | 1.000 | 1.000 | 1.000 | 1.000 | 1.000 | 1.000 | 0.000 |  |  |  |  |  |
| Cbo109 | 1.000 | 1.000 | 1.000 | 1.000 | 1.000 | 1.000 | 1.000 | 1.000 | 0.000 |  |  |  |  |
| Cc04 | 1.000 | 1.000 | 1.000 | 1.000 | 1.000 | 1.000 | 1.000 | 1.000 | 1.000 | 0.000 |  |  |  |
| Cbo151 | 1.000 | 1.000 | 0.437 | 1.000 | 0.287 | 0.448 | 0.511 | 1.000 | 1.000 | 1.000 | 0.000 |  |  |
| Cbo108 | 1.000 | 1.000 | 0.633 | 1.000 | 0.341 | 0.381 | 0.558 | 1.000 | 1.000 | 1.000 | 0.656 | 0.000 |  |
| Cc37 | 1.000 | 1.000 | 1.000 | 1.000 | 1.000 | 1.000 | 1.000 | 1.000 | 1.000 | 1.000 | 1.000 | 1.000 | 0.000 |
