# Supplementary material for: Take one step backward to move forward: Assessment of genetic diversity and population structure of captive Asian woolly-necked storks (Ciconia episcopus)
Source: PLoS One. 2019 Oct 10;14(10):e0223726. doi: 10.1371/journal.pone.0223726 (PMC6786576; doi:10.1371/journal.pone.0223726)
Supplement: S7 Table — Detailed information for all C. episcopus individuals is presented in S1 Table. (DOCX) [file pone.0223726.s007.docx]

**S7 Table.** Observed and expected heterozygosity of *Ciconia episcopus* based on 13 microsatellite loci in each captive breeding. Detailed information for all *C. episcopus* individuals is presented in S1 Table.

| Species | Captivity/wild | *H_o_* | *H_e_* | *p* value |
| --- | --- | --- | --- | --- |
|  |  |  |  |  |
| *Ciconia episcopus* | Khao Kheow Open Zoo | 0.395±0.117 | 0.520±0.213 | *p* < 0.05 |
|  | Nakhon Ratchasima Zoo | 0.613±0.396 | 0.466±0.230 | *p* = 0.1478 |
|  | Dusit Zoo | 0.833±0.258 | 0.639±0.125 | *p* < 0.05 |

Observed heterozygosity (*H_o_*) and Expected heterozygosity (*H_e_*).
