# Supplementary material for: Take one step backward to move forward: Assessment of genetic diversity and population structure of captive Asian woolly-necked storks (Ciconia episcopus)
Source: PLoS One. 2019 Oct 10;14(10):e0223726. doi: 10.1371/journal.pone.0223726 (PMC6786576; doi:10.1371/journal.pone.0223726)
Supplement: S9 Table — Detailed information for all C. episcopus individuals is presented in S1 Table. (DOCX) [file pone.0223726.s009.docx]

**S9 Table.** Pairwise genetic relatedness (*r*) for all 68 *Ciconia episcopus* individuals in Khao Kheow Open Zoo. Detailed information for all *C. episcopus* individuals is presented in S1 Table.

| Individuals pairwise | Pairwise genetic relatedness (*r*) values |
| --- | --- |
| CEP1-CEP2 | 0.048 |
| CEP1-CEP3 | 0.085 |
| CEP2-CEP3 | 0.272 |
| CEP1-CEP4 | 0.048 |
| CEP2-CEP4 | 0.165 |
| CEP3-CEP4 | 0.177 |
| CEP1-CEP5 | 0.070 |
| CEP2-CEP5 | 0.081 |
| CEP3-CEP5 | 0.080 |
| CEP4-CEP5 | 0.091 |
| CEP1-CEP6 | 0.223 |
| CEP2-CEP6 | 0.149 |
| CEP3-CEP6 | 0.187 |
| CEP4-CEP6 | 0.181 |
| CEP5-CEP6 | 0.171 |
| CEP1-CEP7 | 0.131 |
| CEP2-CEP7 | 0.127 |
| CEP3-CEP7 | 0.230 |
| CEP4-CEP7 | 0.159 |
| CEP5-CEP7 | 0.079 |
| CEP6-CEP7 | 0.235 |
| CEP1-CEP8 | 0.146 |
| CEP2-CEP8 | 0.134 |
| CEP3-CEP8 | 0.094 |
| CEP4-CEP8 | 0.107 |
| CEP5-CEP8 | 0.072 |
| CEP6-CEP8 | 0.111 |
| CEP7-CEP8 | 0.190 |
| CEP1-CEP9 | 0.104 |
| CEP2-CEP9 | 0.184 |
| CEP3-CEP9 | 0.326 |
| CEP4-CEP9 | 0.079 |
| CEP5-CEP9 | 0.101 |
| CEP6-CEP9 | 0.169 |
| CEP7-CEP9 | 0.076 |
| CEP8-CEP9 | 0.021 |
| CEP1-CEP10 | 0.081 |
| CEP2-CEP10 | 0.062 |
| CEP3-CEP10 | 0.044 |
| CEP4-CEP10 | 0.055 |
| CEP5-CEP10 | 0.125 |
| CEP6-CEP10 | 0.106 |
| CEP7-CEP10 | 0.013 |
| CEP8-CEP10 | 0.060 |
| CEP9-CEP10 | 0.092 |
| CEP1-CEP11 | 0.048 |
| CEP2-CEP11 | 0.108 |
| CEP3-CEP11 | 0.129 |
| CEP4-CEP11 | 0.190 |
| CEP5-CEP11 | 0.044 |
| CEP6-CEP11 | 0.104 |
| CEP7-CEP11 | 0.082 |
| CEP8-CEP11 | 0.030 |
| CEP9-CEP11 | 0.066 |
| CEP10-CEP11 | 0.074 |
| CEP1-CEP12 | 0.363 |
| CEP2-CEP12 | 0.044 |
| CEP3-CEP12 | 0.033 |
| CEP4-CEP12 | 0.044 |
| CEP5-CEP12 | 0.241 |
| CEP6-CEP12 | 0.153 |
| CEP7-CEP12 | 0.096 |
| CEP8-CEP12 | 0.264 |
| CEP9-CEP12 | 0.063 |
| CEP10-CEP12 | 0.049 |
| CEP11-CEP12 | 0.028 |
| CEP1-CEP13 | 0.034 |
| CEP2-CEP13 | 0.197 |
| CEP3-CEP13 | 0.173 |
| CEP4-CEP13 | 0.170 |
| CEP5-CEP13 | 0.087 |
| CEP6-CEP13 | 0.175 |
| CEP7-CEP13 | 0.152 |
| CEP8-CEP13 | 0.186 |
| CEP9-CEP13 | 0.155 |
| CEP10-CEP13 | 0.075 |
| CEP11-CEP13 | 0.101 |
| CEP12-CEP13 | 0.069 |
| CEP1-CEP14 | -0.025 |
| CEP2-CEP14 | 0.092 |
| CEP3-CEP14 | 0.055 |
| CEP4-CEP14 | 0.164 |
| CEP5-CEP14 | 0.066 |
| CEP6-CEP14 | 0.108 |
| CEP7-CEP14 | 0.085 |
| CEP8-CEP14 | 0.082 |
| CEP9-CEP14 | 0.118 |
| CEP10-CEP14 | 0.030 |
| CEP11-CEP14 | 0.124 |
| CEP12-CEP14 | 0.019 |
| CEP13-CEP14 | 0.167 |
| CEP1-CEP15 | 0.000 |
| CEP2-CEP15 | 0.143 |
| CEP3-CEP15 | 0.080 |
| CEP4-CEP15 | 0.126 |
| CEP5-CEP15 | 0.091 |
| CEP6-CEP15 | 0.101 |
| CEP7-CEP15 | 0.114 |
| CEP8-CEP15 | 0.195 |
| CEP9-CEP15 | 0.031 |
| CEP10-CEP15 | 0.108 |
| CEP11-CEP15 | 0.079 |
| CEP12-CEP15 | 0.079 |
| CEP13-CEP15 | 0.176 |
| CEP14-CEP15 | 0.101 |
| CEP1-CEP16 | 0.078 |
| CEP2-CEP16 | 0.033 |
| CEP3-CEP16 | 0.031 |
| CEP4-CEP16 | 0.042 |
| CEP5-CEP16 | 0.170 |
| CEP6-CEP16 | 0.103 |
| CEP7-CEP16 | 0.011 |
| CEP8-CEP16 | 0.004 |
| CEP9-CEP16 | 0.089 |
| CEP10-CEP16 | 0.133 |
| CEP11-CEP16 | -0.008 |
| CEP12-CEP16 | 0.043 |
| CEP13-CEP16 | 0.019 |
| CEP14-CEP16 | 0.018 |
| CEP15-CEP16 | 0.042 |
| CEP1-CEP17 | -0.085 |
| CEP2-CEP17 | 0.031 |
| CEP3-CEP17 | -0.005 |
| CEP4-CEP17 | 0.104 |
| CEP5-CEP17 | 0.006 |
| CEP6-CEP17 | 0.048 |
| CEP7-CEP17 | 0.060 |
| CEP8-CEP17 | 0.057 |
| CEP9-CEP17 | 0.058 |
| CEP10-CEP17 | -0.030 |
| CEP11-CEP17 | 0.061 |
| CEP12-CEP17 | -0.009 |
| CEP13-CEP17 | 0.107 |
| CEP14-CEP17 | 0.192 |
| CEP15-CEP17 | 0.076 |
| CEP16-CEP17 | -0.040 |
| CEP1-CEP18 | -0.074 |
| CEP2-CEP18 | -0.012 |
| CEP3-CEP18 | -0.005 |
| CEP4-CEP18 | 0.038 |
| CEP5-CEP18 | 0.321 |
| CEP6-CEP18 | 0.003 |
| CEP7-CEP18 | 0.016 |
| CEP8-CEP18 | -0.007 |
| CEP9-CEP18 | 0.007 |
| CEP10-CEP18 | 0.000 |
| CEP11-CEP18 | 0.024 |
| CEP12-CEP18 | -0.037 |
| CEP13-CEP18 | 0.043 |
| CEP14-CEP18 | 0.083 |
| CEP15-CEP18 | 0.042 |
| CEP16-CEP18 | -0.019 |
| CEP17-CEP18 | 0.228 |
| CEP1-CEP19 | -0.014 |
| CEP2-CEP19 | 0.102 |
| CEP3-CEP19 | 0.099 |
| CEP4-CEP19 | 0.175 |
| CEP5-CEP19 | 0.077 |
| CEP6-CEP19 | 0.118 |
| CEP7-CEP19 | 0.162 |
| CEP8-CEP19 | 0.126 |
| CEP9-CEP19 | 0.017 |
| CEP10-CEP19 | 0.041 |
| CEP11-CEP19 | 0.067 |
| CEP12-CEP19 | -0.030 |
| CEP13-CEP19 | 0.108 |
| CEP14-CEP19 | 0.202 |
| CEP15-CEP19 | 0.112 |
| CEP16-CEP19 | 0.088 |
| CEP17-CEP19 | 0.093 |
| CEP18-CEP19 | 0.027 |
| CEP1-CEP20 | -0.014 |
| CEP2-CEP20 | 0.074 |
| CEP3-CEP20 | 0.069 |
| CEP4-CEP20 | 0.048 |
| CEP5-CEP20 | 0.048 |
| CEP6-CEP20 | 0.048 |
| CEP7-CEP20 | 0.126 |
| CEP8-CEP20 | 0.140 |
| CEP9-CEP20 | -0.003 |
| CEP10-CEP20 | 0.075 |
| CEP11-CEP20 | -0.050 |
| CEP12-CEP20 | -0.035 |
| CEP13-CEP20 | 0.087 |
| CEP14-CEP20 | 0.075 |
| CEP15-CEP20 | 0.136 |
| CEP16-CEP20 | 0.079 |
| CEP17-CEP20 | 0.001 |
| CEP18-CEP20 | 0.011 |
| CEP19-CEP20 | 0.179 |
| CEP1-CEP21 | 0.083 |
| CEP2-CEP21 | -0.021 |
| CEP3-CEP21 | -0.049 |
| CEP4-CEP21 | -0.038 |
| CEP5-CEP21 | -0.038 |
| CEP6-CEP21 | -0.028 |
| CEP7-CEP21 | -0.050 |
| CEP8-CEP21 | 0.084 |
| CEP9-CEP21 | 0.051 |
| CEP10-CEP21 | 0.017 |
| CEP11-CEP21 | -0.044 |
| CEP12-CEP21 | 0.088 |
| CEP13-CEP21 | 0.082 |
| CEP14-CEP21 | 0.102 |
| CEP15-CEP21 | 0.016 |
| CEP16-CEP21 | 0.111 |
| CEP17-CEP21 | 0.159 |
| CEP18-CEP21 | 0.117 |
| CEP19-CEP21 | 0.003 |
| CEP20-CEP21 | 0.028 |
| CEP1-CEP22 | -0.083 |
| CEP2-CEP22 | 0.041 |
| CEP3-CEP22 | 0.053 |
| CEP4-CEP22 | 0.033 |
| CEP5-CEP22 | -0.002 |
| CEP6-CEP22 | -0.021 |
| CEP7-CEP22 | 0.124 |
| CEP8-CEP22 | 0.140 |
| CEP9-CEP22 | -0.071 |
| CEP10-CEP22 | 0.045 |
| CEP11-CEP22 | 0.012 |
| CEP12-CEP22 | -0.046 |
| CEP13-CEP22 | 0.054 |
| CEP14-CEP22 | 0.009 |
| CEP15-CEP22 | 0.122 |
| CEP16-CEP22 | -0.028 |
| CEP17-CEP22 | 0.153 |
| CEP18-CEP22 | 0.148 |
| CEP19-CEP22 | 0.088 |
| CEP20-CEP22 | 0.123 |
| CEP21-CEP22 | 0.093 |
| CEP1-CEP23 | -0.015 |
| CEP2-CEP23 | 0.064 |
| CEP3-CEP23 | 0.090 |
| CEP4-CEP23 | 0.031 |
| CEP5-CEP23 | 0.136 |
| CEP6-CEP23 | 0.047 |
| CEP7-CEP23 | -0.046 |
| CEP8-CEP23 | -0.017 |
| CEP9-CEP23 | 0.213 |
| CEP10-CEP23 | 0.059 |
| CEP11-CEP23 | 0.017 |
| CEP12-CEP23 | 0.057 |
| CEP13-CEP23 | 0.068 |
| CEP14-CEP23 | 0.118 |
| CEP15-CEP23 | 0.031 |
| CEP16-CEP23 | 0.040 |
| CEP17-CEP23 | 0.228 |
| CEP18-CEP23 | 0.180 |
| CEP19-CEP23 | 0.020 |
| CEP20-CEP23 | -0.034 |
| CEP21-CEP23 | 0.149 |
| CEP22-CEP23 | 0.137 |
| CEP1-CEP24 | 0.000 |
| CEP2-CEP24 | 0.101 |
| CEP3-CEP24 | 0.090 |
| CEP4-CEP24 | 0.101 |
| CEP5-CEP24 | 0.228 |
| CEP6-CEP24 | 0.140 |
| CEP7-CEP24 | 0.153 |
| CEP8-CEP24 | 0.146 |
| CEP9-CEP24 | 0.050 |
| CEP10-CEP24 | 0.036 |
| CEP11-CEP24 | 0.025 |
| CEP12-CEP24 | 0.245 |
| CEP13-CEP24 | 0.126 |
| CEP14-CEP24 | 0.129 |
| CEP15-CEP24 | 0.136 |
| CEP16-CEP24 | 0.090 |
| CEP17-CEP24 | 0.051 |
| CEP18-CEP24 | 0.023 |
| CEP19-CEP24 | 0.139 |
| CEP20-CEP24 | 0.135 |
| CEP21-CEP24 | 0.025 |
| CEP22-CEP24 | 0.014 |
| CEP23-CEP24 | 0.047 |
| CEP1-CEP25 | 0.105 |
| CEP2-CEP25 | 0.095 |
| CEP3-CEP25 | 0.080 |
| CEP4-CEP25 | 0.091 |
| CEP5-CEP25 | 0.091 |
| CEP6-CEP25 | 0.153 |
| CEP7-CEP25 | 0.166 |
| CEP8-CEP25 | 0.134 |
| CEP9-CEP25 | 0.031 |
| CEP10-CEP25 | 0.092 |
| CEP11-CEP25 | 0.044 |
| CEP12-CEP25 | 0.079 |
| CEP13-CEP25 | 0.114 |
| CEP14-CEP25 | 0.066 |
| CEP15-CEP25 | 0.153 |
| CEP16-CEP25 | 0.042 |
| CEP17-CEP25 | 0.041 |
| CEP18-CEP25 | 0.042 |
| CEP19-CEP25 | 0.077 |
| CEP20-CEP25 | 0.109 |
| CEP21-CEP25 | -0.011 |
| CEP22-CEP25 | 0.060 |
| CEP23-CEP25 | -0.004 |
| CEP24-CEP25 | 0.136 |
| CEP1-CEP26 | -0.025 |
| CEP2-CEP26 | 0.072 |
| CEP3-CEP26 | 0.045 |
| CEP4-CEP26 | 0.091 |
| CEP5-CEP26 | 0.183 |
| CEP6-CEP26 | 0.037 |
| CEP7-CEP26 | 0.015 |
| CEP8-CEP26 | 0.043 |
| CEP9-CEP26 | 0.099 |
| CEP10-CEP26 | 0.049 |
| CEP11-CEP26 | 0.080 |
| CEP12-CEP26 | 0.107 |
| CEP13-CEP26 | 0.128 |
| CEP14-CEP26 | 0.231 |
| CEP15-CEP26 | 0.091 |
| CEP16-CEP26 | 0.084 |
| CEP17-CEP26 | 0.118 |
| CEP18-CEP26 | 0.071 |
| CEP19-CEP26 | 0.129 |
| CEP20-CEP26 | 0.075 |
| CEP21-CEP26 | 0.092 |
| CEP22-CEP26 | 0.028 |
| CEP23-CEP26 | 0.207 |
| CEP24-CEP26 | 0.217 |
| CEP25-CEP26 | 0.056 |
| CEP1-CEP27 | -0.075 |
| CEP2-CEP27 | -0.015 |
| CEP3-CEP27 | -0.009 |
| CEP4-CEP27 | 0.004 |
| CEP5-CEP27 | 0.109 |
| CEP6-CEP27 | -0.050 |
| CEP7-CEP27 | -0.006 |
| CEP8-CEP27 | -0.011 |
| CEP9-CEP27 | 0.007 |
| CEP10-CEP27 | 0.000 |
| CEP11-CEP27 | 0.028 |
| CEP12-CEP27 | 0.030 |
| CEP13-CEP27 | 0.041 |
| CEP14-CEP27 | 0.050 |
| CEP15-CEP27 | 0.004 |
| CEP16-CEP27 | 0.138 |
| CEP17-CEP27 | 0.104 |
| CEP18-CEP27 | 0.099 |
| CEP19-CEP27 | 0.026 |
| CEP20-CEP27 | -0.028 |
| CEP21-CEP27 | 0.220 |
| CEP22-CEP27 | 0.122 |
| CEP23-CEP27 | 0.263 |
| CEP24-CEP27 | 0.090 |
| CEP25-CEP27 | -0.030 |
| CEP26-CEP27 | 0.209 |
| CEP1-CEP28 | -0.049 |
| CEP2-CEP28 | 0.011 |
| CEP3-CEP28 | -0.036 |
| CEP4-CEP28 | 0.011 |
| CEP5-CEP28 | 0.103 |
| CEP6-CEP28 | -0.024 |
| CEP7-CEP28 | -0.012 |
| CEP8-CEP28 | 0.016 |
| CEP9-CEP28 | -0.038 |
| CEP10-CEP28 | -0.013 |
| CEP11-CEP28 | 0.008 |
| CEP12-CEP28 | 0.078 |
| CEP13-CEP28 | -0.003 |
| CEP14-CEP28 | -0.014 |
| CEP15-CEP28 | 0.046 |
| CEP16-CEP28 | 0.044 |
| CEP17-CEP28 | 0.021 |
| CEP18-CEP28 | -0.003 |
| CEP19-CEP28 | -0.001 |
| CEP20-CEP28 | 0.048 |
| CEP21-CEP28 | -0.056 |
| CEP22-CEP28 | 0.023 |
| CEP23-CEP28 | 0.056 |
| CEP24-CEP28 | 0.138 |
| CEP25-CEP28 | 0.011 |
| CEP26-CEP28 | 0.113 |
| CEP27-CEP28 | 0.136 |
| CEP1-CEP29 | -0.049 |
| CEP2-CEP29 | -0.011 |
| CEP3-CEP29 | -0.017 |
| CEP4-CEP29 | 0.026 |
| CEP5-CEP29 | -0.005 |
| CEP6-CEP29 | -0.009 |
| CEP7-CEP29 | 0.004 |
| CEP8-CEP29 | 0.008 |
| CEP9-CEP29 | -0.038 |
| CEP10-CEP29 | 0.052 |
| CEP11-CEP29 | 0.042 |
| CEP12-CEP29 | -0.049 |
| CEP13-CEP29 | -0.012 |
| CEP14-CEP29 | 0.054 |
| CEP15-CEP29 | 0.056 |
| CEP16-CEP29 | 0.006 |
| CEP17-CEP29 | 0.091 |
| CEP18-CEP29 | 0.105 |
| CEP19-CEP29 | 0.067 |
| CEP20-CEP29 | 0.079 |
| CEP21-CEP29 | 0.097 |
| CEP22-CEP29 | 0.108 |
| CEP23-CEP29 | 0.044 |
| CEP24-CEP29 | 0.063 |
| CEP25-CEP29 | 0.043 |
| CEP26-CEP29 | 0.041 |
| CEP27-CEP29 | 0.117 |
| CEP28-CEP29 | 0.022 |
| CEP1-CEP30 | 0.142 |
| CEP2-CEP30 | 0.117 |
| CEP3-CEP30 | 0.259 |
| CEP4-CEP30 | 0.012 |
| CEP5-CEP30 | -0.037 |
| CEP6-CEP30 | 0.031 |
| CEP7-CEP30 | 0.009 |
| CEP8-CEP30 | 0.041 |
| CEP9-CEP30 | 0.593 |
| CEP10-CEP30 | -0.045 |
| CEP11-CEP30 | -0.004 |
| CEP12-CEP30 | 0.098 |
| CEP13-CEP30 | 0.087 |
| CEP14-CEP30 | 0.061 |
| CEP15-CEP30 | -0.037 |
| CEP16-CEP30 | -0.045 |
| CEP17-CEP30 | 0.008 |
| CEP18-CEP30 | 0.000 |
| CEP19-CEP30 | 0.005 |
| CEP20-CEP30 | -0.015 |
| CEP21-CEP30 | 0.142 |
| CEP22-CEP30 | -0.079 |
| CEP23-CEP30 | 0.094 |
| CEP24-CEP30 | 0.035 |
| CEP25-CEP30 | -0.037 |
| CEP26-CEP30 | 0.041 |
| CEP27-CEP30 | -0.001 |
| CEP28-CEP30 | 0.206 |
| CEP29-CEP30 | 0.007 |
| CEP1-CEP31 | -0.060 |
| CEP2-CEP31 | 0.033 |
| CEP3-CEP31 | -0.017 |
| CEP4-CEP31 | 0.060 |
| CEP5-CEP31 | -0.006 |
| CEP6-CEP31 | 0.020 |
| CEP7-CEP31 | -0.002 |
| CEP8-CEP31 | 0.037 |
| CEP9-CEP31 | 0.013 |
| CEP10-CEP31 | 0.022 |
| CEP11-CEP31 | 0.047 |
| CEP12-CEP31 | -0.056 |
| CEP13-CEP31 | 0.052 |
| CEP14-CEP31 | 0.078 |
| CEP15-CEP31 | 0.056 |
| CEP16-CEP31 | 0.143 |
| CEP17-CEP31 | 0.133 |
| CEP18-CEP31 | 0.040 |
| CEP19-CEP31 | 0.049 |
| CEP20-CEP31 | -0.020 |
| CEP21-CEP31 | 0.244 |
| CEP22-CEP31 | 0.078 |
| CEP23-CEP31 | 0.091 |
| CEP24-CEP31 | 0.004 |
| CEP25-CEP31 | 0.007 |
| CEP26-CEP31 | 0.037 |
| CEP27-CEP31 | 0.190 |
| CEP28-CEP31 | 0.137 |
| CEP29-CEP31 | 0.051 |
| CEP30-CEP31 | 0.089 |
| CEP1-CEP32 | 0.097 |
| CEP2-CEP32 | -0.005 |
| CEP3-CEP32 | -0.045 |
| CEP4-CEP32 | 0.032 |
| CEP5-CEP32 | -0.034 |
| CEP6-CEP32 | -0.037 |
| CEP7-CEP32 | -0.060 |
| CEP8-CEP32 | 0.067 |
| CEP9-CEP32 | -0.024 |
| CEP10-CEP32 | 0.023 |
| CEP11-CEP32 | 0.048 |
| CEP12-CEP32 | 0.062 |
| CEP13-CEP32 | -0.005 |
| CEP14-CEP32 | 0.102 |
| CEP15-CEP32 | 0.028 |
| CEP16-CEP32 | -0.023 |
| CEP17-CEP32 | 0.105 |
| CEP18-CEP32 | 0.041 |
| CEP19-CEP32 | 0.074 |
| CEP20-CEP32 | 0.015 |
| CEP21-CEP32 | 0.198 |
| CEP22-CEP32 | 0.079 |
| CEP23-CEP32 | 0.092 |
| CEP24-CEP32 | -0.001 |
| CEP25-CEP32 | -0.021 |
| CEP26-CEP32 | 0.090 |
| CEP27-CEP32 | 0.033 |
| CEP28-CEP32 | 0.119 |
| CEP29-CEP32 | 0.105 |
| CEP30-CEP32 | 0.192 |
| CEP31-CEP32 | 0.163 |
| CEP1-CEP33 | 0.021 |
| CEP2-CEP33 | 0.015 |
| CEP3-CEP33 | 0.004 |
| CEP4-CEP33 | 0.015 |
| CEP5-CEP33 | 0.199 |
| CEP6-CEP33 | 0.124 |
| CEP7-CEP33 | 0.067 |
| CEP8-CEP33 | 0.060 |
| CEP9-CEP33 | 0.071 |
| CEP10-CEP33 | 0.057 |
| CEP11-CEP33 | -0.027 |
| CEP12-CEP33 | 0.214 |
| CEP13-CEP33 | 0.040 |
| CEP14-CEP33 | -0.010 |
| CEP15-CEP33 | 0.050 |
| CEP16-CEP33 | 0.171 |
| CEP17-CEP33 | 0.025 |
| CEP18-CEP33 | -0.003 |
| CEP19-CEP33 | 0.004 |
| CEP20-CEP33 | -0.001 |
| CEP21-CEP33 | -0.017 |
| CEP22-CEP33 | -0.012 |
| CEP23-CEP33 | 0.021 |
| CEP24-CEP33 | 0.204 |
| CEP25-CEP33 | 0.050 |
| CEP26-CEP33 | 0.065 |
| CEP27-CEP33 | -0.039 |
| CEP28-CEP33 | 0.388 |
| CEP29-CEP33 | 0.022 |
| CEP30-CEP33 | 0.246 |
| CEP31-CEP33 | 0.141 |
| CEP32-CEP33 | 0.084 |
| CEP1-CEP34 | -0.060 |
| CEP2-CEP34 | 0.123 |
| CEP3-CEP34 | -0.046 |
| CEP4-CEP34 | 0.000 |
| CEP5-CEP34 | -0.035 |
| CEP6-CEP34 | -0.035 |
| CEP7-CEP34 | -0.023 |
| CEP8-CEP34 | 0.059 |
| CEP9-CEP34 | -0.006 |
| CEP10-CEP34 | 0.029 |
| CEP11-CEP34 | -0.003 |
| CEP12-CEP34 | -0.060 |
| CEP13-CEP34 | 0.039 |
| CEP14-CEP34 | 0.017 |
| CEP15-CEP34 | 0.088 |
| CEP16-CEP34 | -0.024 |
| CEP17-CEP34 | 0.107 |
| CEP18-CEP34 | 0.041 |
| CEP19-CEP34 | -0.011 |
| CEP20-CEP34 | 0.042 |
| CEP21-CEP34 | 0.084 |
| CEP22-CEP34 | 0.121 |
| CEP23-CEP34 | 0.072 |
| CEP24-CEP34 | 0.000 |
| CEP25-CEP34 | 0.027 |
| CEP26-CEP34 | 0.017 |
| CEP27-CEP34 | 0.103 |
| CEP28-CEP34 | 0.046 |
| CEP29-CEP34 | 0.155 |
| CEP30-CEP34 | -0.056 |
| CEP31-CEP34 | 0.097 |
| CEP32-CEP34 | 0.079 |
| CEP33-CEP34 | 0.011 |
| CEP1-CEP35 | -0.060 |
| CEP2-CEP35 | 0.062 |
| CEP3-CEP35 | -0.046 |
| CEP4-CEP35 | -0.035 |
| CEP5-CEP35 | 0.092 |
| CEP6-CEP35 | -0.035 |
| CEP7-CEP35 | -0.023 |
| CEP8-CEP35 | -0.029 |
| CEP9-CEP35 | -0.006 |
| CEP10-CEP35 | -0.024 |
| CEP11-CEP35 | -0.038 |
| CEP12-CEP35 | 0.067 |
| CEP13-CEP35 | -0.049 |
| CEP14-CEP35 | 0.035 |
| CEP15-CEP35 | 0.000 |
| CEP16-CEP35 | 0.033 |
| CEP17-CEP35 | 0.072 |
| CEP18-CEP35 | 0.041 |
| CEP19-CEP35 | 0.006 |
| CEP20-CEP35 | 0.041 |
| CEP21-CEP35 | 0.083 |
| CEP22-CEP35 | 0.032 |
| CEP23-CEP35 | 0.107 |
| CEP24-CEP35 | 0.179 |
| CEP25-CEP35 | 0.000 |
| CEP26-CEP35 | 0.162 |
| CEP27-CEP35 | 0.208 |
| CEP28-CEP35 | 0.138 |
| CEP29-CEP35 | 0.181 |
| CEP30-CEP35 | -0.004 |
| CEP31-CEP35 | 0.035 |
| CEP32-CEP35 | 0.069 |
| CEP33-CEP35 | 0.125 |
| CEP34-CEP35 | 0.354 |
| CEP1-CEP36 | -0.049 |
| CEP2-CEP36 | 0.099 |
| CEP3-CEP36 | -0.036 |
| CEP4-CEP36 | -0.024 |
| CEP5-CEP36 | -0.024 |
| CEP6-CEP36 | -0.024 |
| CEP7-CEP36 | -0.012 |
| CEP8-CEP36 | 0.035 |
| CEP9-CEP36 | -0.038 |
| CEP10-CEP36 | 0.040 |
| CEP11-CEP36 | -0.027 |
| CEP12-CEP36 | -0.049 |
| CEP13-CEP36 | 0.015 |
| CEP14-CEP36 | 0.003 |
| CEP15-CEP36 | 0.064 |
| CEP16-CEP36 | -0.013 |
| CEP17-CEP36 | 0.041 |
| CEP18-CEP36 | 0.051 |
| CEP19-CEP36 | 0.017 |
| CEP20-CEP36 | 0.105 |
| CEP21-CEP36 | 0.105 |
| CEP22-CEP36 | 0.097 |
| CEP23-CEP36 | 0.006 |
| CEP24-CEP36 | 0.063 |
| CEP25-CEP36 | 0.037 |
| CEP26-CEP36 | 0.003 |
| CEP27-CEP36 | -0.011 |
| CEP28-CEP36 | 0.148 |
| CEP29-CEP36 | 0.128 |
| CEP30-CEP36 | 0.133 |
| CEP31-CEP36 | 0.093 |
| CEP32-CEP36 | 0.127 |
| CEP33-CEP36 | 0.148 |
| CEP34-CEP36 | 0.286 |
| CEP35-CEP36 | 0.284 |
| CEP1-CEP37 | -0.012 |
| CEP2-CEP37 | 0.110 |
| CEP3-CEP37 | 0.083 |
| CEP4-CEP37 | 0.013 |
| CEP5-CEP37 | 0.079 |
| CEP6-CEP37 | 0.013 |
| CEP7-CEP37 | 0.091 |
| CEP8-CEP37 | 0.003 |
| CEP9-CEP37 | -0.001 |
| CEP10-CEP37 | -0.025 |
| CEP11-CEP37 | 0.010 |
| CEP12-CEP37 | 0.054 |
| CEP13-CEP37 | -0.001 |
| CEP14-CEP37 | -0.060 |
| CEP15-CEP37 | -0.001 |
| CEP16-CEP37 | 0.090 |
| CEP17-CEP37 | 0.029 |
| CEP18-CEP37 | 0.040 |
| CEP19-CEP37 | -0.014 |
| CEP20-CEP37 | 0.021 |
| CEP21-CEP37 | -0.013 |
| CEP22-CEP37 | 0.098 |
| CEP23-CEP37 | -0.006 |
| CEP24-CEP37 | 0.114 |
| CEP25-CEP37 | -0.001 |
| CEP26-CEP37 | 0.054 |
| CEP27-CEP37 | 0.011 |
| CEP28-CEP37 | 0.251 |
| CEP29-CEP37 | 0.038 |
| CEP30-CEP37 | 0.118 |
| CEP31-CEP37 | 0.055 |
| CEP32-CEP37 | 0.037 |
| CEP33-CEP37 | 0.365 |
| CEP34-CEP37 | 0.221 |
| CEP35-CEP37 | 0.335 |
| CEP36-CEP37 | 0.295 |
| CEP1-CEP38 | -0.049 |
| CEP2-CEP38 | 0.086 |
| CEP3-CEP38 | -0.036 |
| CEP4-CEP38 | 0.007 |
| CEP5-CEP38 | -0.024 |
| CEP6-CEP38 | -0.009 |
| CEP7-CEP38 | 0.004 |
| CEP8-CEP38 | 0.008 |
| CEP9-CEP38 | -0.038 |
| CEP10-CEP38 | 0.013 |
| CEP11-CEP38 | 0.004 |
| CEP12-CEP38 | -0.049 |
| CEP13-CEP38 | -0.012 |
| CEP14-CEP38 | 0.035 |
| CEP15-CEP38 | 0.037 |
| CEP16-CEP38 | -0.013 |
| CEP17-CEP38 | 0.017 |
| CEP18-CEP38 | 0.012 |
| CEP19-CEP38 | 0.048 |
| CEP20-CEP38 | 0.079 |
| CEP21-CEP38 | 0.023 |
| CEP22-CEP38 | 0.015 |
| CEP23-CEP38 | -0.049 |
| CEP24-CEP38 | 0.063 |
| CEP25-CEP38 | 0.024 |
| CEP26-CEP38 | 0.003 |
| CEP27-CEP38 | 0.141 |
| CEP28-CEP38 | 0.022 |
| CEP29-CEP38 | 0.283 |
| CEP30-CEP38 | 0.007 |
| CEP31-CEP38 | 0.005 |
| CEP32-CEP38 | 0.039 |
| CEP33-CEP38 | 0.022 |
| CEP34-CEP38 | 0.411 |
| CEP35-CEP38 | 0.437 |
| CEP36-CEP38 | 0.295 |
| CEP37-CEP38 | 0.204 |
| CEP1-CEP39 | -0.001 |
| CEP2-CEP39 | 0.183 |
| CEP3-CEP39 | 0.061 |
| CEP4-CEP39 | 0.059 |
| CEP5-CEP39 | -0.024 |
| CEP6-CEP39 | 0.024 |
| CEP7-CEP39 | 0.002 |
| CEP8-CEP39 | 0.035 |
| CEP9-CEP39 | 0.010 |
| CEP10-CEP39 | 0.040 |
| CEP11-CEP39 | 0.056 |
| CEP12-CEP39 | -0.084 |
| CEP13-CEP39 | 0.099 |
| CEP14-CEP39 | -0.014 |
| CEP15-CEP39 | 0.064 |
| CEP16-CEP39 | -0.013 |
| CEP17-CEP39 | 0.095 |
| CEP18-CEP39 | 0.071 |
| CEP19-CEP39 | -0.001 |
| CEP20-CEP39 | 0.018 |
| CEP21-CEP39 | 0.107 |
| CEP22-CEP39 | 0.151 |
| CEP23-CEP39 | 0.095 |
| CEP24-CEP39 | -0.024 |
| CEP25-CEP39 | 0.002 |
| CEP26-CEP39 | -0.014 |
| CEP27-CEP39 | 0.051 |
| CEP28-CEP39 | 0.022 |
| CEP29-CEP39 | 0.068 |
| CEP30-CEP39 | 0.003 |
| CEP31-CEP39 | 0.092 |
| CEP32-CEP39 | 0.074 |
| CEP33-CEP39 | -0.013 |
| CEP34-CEP39 | 0.313 |
| CEP35-CEP39 | 0.224 |
| CEP36-CEP39 | 0.289 |
| CEP37-CEP39 | 0.272 |
| CEP38-CEP39 | 0.207 |
| CEP1-CEP40 | -0.119 |
| CEP2-CEP40 | -0.117 |
| CEP3-CEP40 | -0.122 |
| CEP4-CEP40 | -0.079 |
| CEP5-CEP40 | -0.111 |
| CEP6-CEP40 | -0.114 |
| CEP7-CEP40 | -0.137 |
| CEP8-CEP40 | -0.114 |
| CEP9-CEP40 | -0.137 |
| CEP10-CEP40 | -0.033 |
| CEP11-CEP40 | -0.056 |
| CEP12-CEP40 | -0.142 |
| CEP13-CEP40 | -0.110 |
| CEP14-CEP40 | -0.097 |
| CEP15-CEP40 | -0.084 |
| CEP16-CEP40 | -0.100 |
| CEP17-CEP40 | -0.097 |
| CEP18-CEP40 | -0.083 |
| CEP19-CEP40 | -0.091 |
| CEP20-CEP40 | -0.114 |
| CEP21-CEP40 | -0.091 |
| CEP22-CEP40 | -0.087 |
| CEP23-CEP40 | -0.110 |
| CEP24-CEP40 | -0.130 |
| CEP25-CEP40 | -0.077 |
| CEP26-CEP40 | -0.110 |
| CEP27-CEP40 | -0.099 |
| CEP28-CEP40 | -0.119 |
| CEP29-CEP40 | -0.052 |
| CEP30-CEP40 | -0.126 |
| CEP31-CEP40 | -0.082 |
| CEP32-CEP40 | -0.062 |
| CEP33-CEP40 | -0.119 |
| CEP34-CEP40 | -0.103 |
| CEP35-CEP40 | -0.130 |
| CEP36-CEP40 | -0.092 |
| CEP37-CEP40 | -0.130 |
| CEP38-CEP40 | -0.090 |
| CEP39-CEP40 | -0.092 |
| CEP1-CEP41 | -0.118 |
| CEP2-CEP41 | -0.077 |
| CEP3-CEP41 | -0.101 |
| CEP4-CEP41 | -0.059 |
| CEP5-CEP41 | -0.090 |
| CEP6-CEP41 | -0.035 |
| CEP7-CEP41 | -0.057 |
| CEP8-CEP41 | -0.035 |
| CEP9-CEP41 | -0.091 |
| CEP10-CEP41 | -0.071 |
| CEP11-CEP41 | -0.094 |
| CEP12-CEP41 | -0.063 |
| CEP13-CEP41 | -0.031 |
| CEP14-CEP41 | -0.071 |
| CEP15-CEP41 | -0.063 |
| CEP16-CEP41 | -0.118 |
| CEP17-CEP41 | -0.071 |
| CEP18-CEP41 | -0.121 |
| CEP19-CEP41 | -0.070 |
| CEP20-CEP41 | -0.114 |
| CEP21-CEP41 | -0.065 |
| CEP22-CEP41 | -0.125 |
| CEP23-CEP41 | -0.142 |
| CEP24-CEP41 | -0.051 |
| CEP25-CEP41 | -0.056 |
| CEP26-CEP41 | -0.142 |
| CEP27-CEP41 | -0.137 |
| CEP28-CEP41 | -0.118 |
| CEP29-CEP41 | -0.089 |
| CEP30-CEP41 | -0.086 |
| CEP31-CEP41 | -0.055 |
| CEP32-CEP41 | -0.095 |
| CEP33-CEP41 | -0.040 |
| CEP34-CEP41 | -0.097 |
| CEP35-CEP41 | -0.124 |
| CEP36-CEP41 | -0.092 |
| CEP37-CEP41 | -0.130 |
| CEP38-CEP41 | -0.089 |
| CEP39-CEP41 | -0.092 |
| CEP40-CEP41 | 0.128 |
| CEP1-CEP42 | -0.011 |
| CEP2-CEP42 | -0.114 |
| CEP3-CEP42 | -0.086 |
| CEP4-CEP42 | -0.108 |
| CEP5-CEP42 | -0.108 |
| CEP6-CEP42 | -0.075 |
| CEP7-CEP42 | -0.031 |
| CEP8-CEP42 | -0.079 |
| CEP9-CEP42 | -0.141 |
| CEP10-CEP42 | -0.030 |
| CEP11-CEP42 | -0.092 |
| CEP12-CEP42 | -0.139 |
| CEP13-CEP42 | -0.114 |
| CEP14-CEP42 | -0.133 |
| CEP15-CEP42 | -0.081 |
| CEP16-CEP42 | -0.097 |
| CEP17-CEP42 | -0.133 |
| CEP18-CEP42 | -0.103 |
| CEP19-CEP42 | -0.086 |
| CEP20-CEP42 | -0.079 |
| CEP21-CEP42 | -0.095 |
| CEP22-CEP42 | -0.019 |
| CEP23-CEP42 | -0.114 |
| CEP24-CEP42 | -0.127 |
| CEP25-CEP42 | -0.021 |
| CEP26-CEP42 | -0.114 |
| CEP27-CEP42 | -0.070 |
| CEP28-CEP42 | -0.116 |
| CEP29-CEP42 | -0.065 |
| CEP30-CEP42 | -0.130 |
| CEP31-CEP42 | -0.094 |
| CEP32-CEP42 | -0.075 |
| CEP33-CEP42 | -0.116 |
| CEP34-CEP42 | -0.100 |
| CEP35-CEP42 | -0.127 |
| CEP36-CEP42 | -0.089 |
| CEP37-CEP42 | -0.094 |
| CEP38-CEP42 | -0.103 |
| CEP39-CEP42 | -0.089 |
| CEP40-CEP42 | 0.095 |
| CEP41-CEP42 | 0.062 |
| CEP1-CEP43 | -0.141 |
| CEP2-CEP43 | -0.119 |
| CEP3-CEP43 | -0.068 |
| CEP4-CEP43 | -0.092 |
| CEP5-CEP43 | -0.123 |
| CEP6-CEP43 | -0.097 |
| CEP7-CEP43 | 0.012 |
| CEP8-CEP43 | -0.031 |
| CEP9-CEP43 | -0.139 |
| CEP10-CEP43 | -0.074 |
| CEP11-CEP43 | -0.098 |
| CEP12-CEP43 | -0.125 |
| CEP13-CEP43 | -0.093 |
| CEP14-CEP43 | -0.109 |
| CEP15-CEP43 | -0.096 |
| CEP16-CEP43 | -0.132 |
| CEP17-CEP43 | -0.109 |
| CEP18-CEP43 | -0.124 |
| CEP19-CEP43 | -0.037 |
| CEP20-CEP43 | -0.071 |
| CEP21-CEP43 | -0.103 |
| CEP22-CEP43 | 0.003 |
| CEP23-CEP43 | -0.151 |
| CEP24-CEP43 | -0.113 |
| CEP25-CEP43 | -0.089 |
| CEP26-CEP43 | -0.151 |
| CEP27-CEP43 | -0.074 |
| CEP28-CEP43 | -0.141 |
| CEP29-CEP43 | -0.093 |
| CEP30-CEP43 | -0.129 |
| CEP31-CEP43 | -0.094 |
| CEP32-CEP43 | -0.104 |
| CEP33-CEP43 | -0.102 |
| CEP34-CEP43 | -0.125 |
| CEP35-CEP43 | -0.152 |
| CEP36-CEP43 | -0.114 |
| CEP37-CEP43 | -0.087 |
| CEP38-CEP43 | -0.112 |
| CEP39-CEP43 | -0.114 |
| CEP40-CEP43 | 0.161 |
| CEP41-CEP43 | 0.187 |
| CEP42-CEP43 | 0.161 |
| CEP1-CEP44 | -0.036 |
| CEP2-CEP44 | -0.139 |
| CEP3-CEP44 | -0.078 |
| CEP4-CEP44 | -0.133 |
| CEP5-CEP44 | -0.133 |
| CEP6-CEP44 | -0.100 |
| CEP7-CEP44 | 0.010 |
| CEP8-CEP44 | -0.071 |
| CEP9-CEP44 | -0.159 |
| CEP10-CEP44 | -0.055 |
| CEP11-CEP44 | -0.110 |
| CEP12-CEP44 | -0.164 |
| CEP13-CEP44 | -0.133 |
| CEP14-CEP44 | -0.151 |
| CEP15-CEP44 | -0.106 |
| CEP16-CEP44 | -0.122 |
| CEP17-CEP44 | -0.151 |
| CEP18-CEP44 | -0.121 |
| CEP19-CEP44 | -0.078 |
| CEP20-CEP44 | -0.071 |
| CEP21-CEP44 | -0.113 |
| CEP22-CEP44 | 0.022 |
| CEP23-CEP44 | -0.132 |
| CEP24-CEP44 | -0.152 |
| CEP25-CEP44 | -0.046 |
| CEP26-CEP44 | -0.132 |
| CEP27-CEP44 | -0.055 |
| CEP28-CEP44 | -0.141 |
| CEP29-CEP44 | -0.090 |
| CEP30-CEP44 | -0.148 |
| CEP31-CEP44 | -0.120 |
| CEP32-CEP44 | -0.100 |
| CEP33-CEP44 | -0.141 |
| CEP34-CEP44 | -0.125 |
| CEP35-CEP44 | -0.152 |
| CEP36-CEP44 | -0.114 |
| CEP37-CEP44 | -0.087 |
| CEP38-CEP44 | -0.128 |
| CEP39-CEP44 | -0.114 |
| CEP40-CEP44 | 0.063 |
| CEP41-CEP44 | 0.062 |
| CEP42-CEP44 | 0.292 |
| CEP43-CEP44 | 0.196 |
| CEP1-CEP45 | -0.036 |
| CEP2-CEP45 | -0.139 |
| CEP3-CEP45 | -0.078 |
| CEP4-CEP45 | -0.133 |
| CEP5-CEP45 | -0.133 |
| CEP6-CEP45 | -0.100 |
| CEP7-CEP45 | 0.010 |
| CEP8-CEP45 | -0.071 |
| CEP9-CEP45 | -0.159 |
| CEP10-CEP45 | -0.055 |
| CEP11-CEP45 | -0.110 |
| CEP12-CEP45 | -0.164 |
| CEP13-CEP45 | -0.133 |
| CEP14-CEP45 | -0.151 |
| CEP15-CEP45 | -0.106 |
| CEP16-CEP45 | -0.122 |
| CEP17-CEP45 | -0.151 |
| CEP18-CEP45 | -0.121 |
| CEP19-CEP45 | -0.078 |
| CEP20-CEP45 | -0.071 |
| CEP21-CEP45 | -0.113 |
| CEP22-CEP45 | 0.022 |
| CEP23-CEP45 | -0.132 |
| CEP24-CEP45 | -0.152 |
| CEP25-CEP45 | -0.046 |
| CEP26-CEP45 | -0.132 |
| CEP27-CEP45 | -0.055 |
| CEP28-CEP45 | -0.141 |
| CEP29-CEP45 | -0.090 |
| CEP30-CEP45 | -0.148 |
| CEP31-CEP45 | -0.120 |
| CEP32-CEP45 | -0.100 |
| CEP33-CEP45 | -0.141 |
| CEP34-CEP45 | -0.125 |
| CEP35-CEP45 | -0.152 |
| CEP36-CEP45 | -0.114 |
| CEP37-CEP45 | -0.087 |
| CEP38-CEP45 | -0.128 |
| CEP39-CEP45 | -0.114 |
| CEP40-CEP45 | 0.063 |
| CEP41-CEP45 | 0.062 |
| CEP42-CEP45 | 0.292 |
| CEP43-CEP45 | 0.196 |
| CEP44-CEP45 | 0.567 |
| CEP1-CEP46 | -0.119 |
| CEP2-CEP46 | -0.097 |
| CEP3-CEP46 | -0.122 |
| CEP4-CEP46 | -0.079 |
| CEP5-CEP46 | -0.110 |
| CEP6-CEP46 | -0.075 |
| CEP7-CEP46 | -0.097 |
| CEP8-CEP46 | -0.075 |
| CEP9-CEP46 | -0.117 |
| CEP10-CEP46 | -0.071 |
| CEP11-CEP46 | -0.095 |
| CEP12-CEP46 | -0.103 |
| CEP13-CEP46 | -0.071 |
| CEP14-CEP46 | -0.097 |
| CEP15-CEP46 | -0.084 |
| CEP16-CEP46 | -0.119 |
| CEP17-CEP46 | -0.097 |
| CEP18-CEP46 | -0.121 |
| CEP19-CEP46 | -0.090 |
| CEP20-CEP46 | -0.066 |
| CEP21-CEP46 | -0.090 |
| CEP22-CEP46 | -0.126 |
| CEP23-CEP46 | -0.148 |
| CEP24-CEP46 | -0.091 |
| CEP25-CEP46 | -0.076 |
| CEP26-CEP46 | -0.148 |
| CEP27-CEP46 | -0.137 |
| CEP28-CEP46 | -0.071 |
| CEP29-CEP46 | -0.090 |
| CEP30-CEP46 | -0.106 |
| CEP31-CEP46 | -0.081 |
| CEP32-CEP46 | -0.101 |
| CEP33-CEP46 | -0.080 |
| CEP34-CEP46 | -0.103 |
| CEP35-CEP46 | -0.130 |
| CEP36-CEP46 | -0.092 |
| CEP37-CEP46 | -0.130 |
| CEP38-CEP46 | -0.090 |
| CEP39-CEP46 | -0.092 |
| CEP40-CEP46 | 0.133 |
| CEP41-CEP46 | 0.209 |
| CEP42-CEP46 | 0.067 |
| CEP43-CEP46 | 0.173 |
| CEP44-CEP46 | 0.067 |
| CEP45-CEP46 | 0.067 |
| CEP1-CEP47 | -0.028 |
| CEP2-CEP47 | -0.144 |
| CEP3-CEP47 | -0.155 |
| CEP4-CEP47 | -0.113 |
| CEP5-CEP47 | -0.144 |
| CEP6-CEP47 | -0.076 |
| CEP7-CEP47 | -0.098 |
| CEP8-CEP47 | -0.155 |
| CEP9-CEP47 | -0.144 |
| CEP10-CEP47 | -0.112 |
| CEP11-CEP47 | -0.101 |
| CEP12-CEP47 | -0.156 |
| CEP13-CEP47 | -0.144 |
| CEP14-CEP47 | -0.123 |
| CEP15-CEP47 | -0.144 |
| CEP16-CEP47 | -0.133 |
| CEP17-CEP47 | -0.123 |
| CEP18-CEP47 | -0.128 |
| CEP19-CEP47 | -0.124 |
| CEP20-CEP47 | -0.058 |
| CEP21-CEP47 | -0.144 |
| CEP22-CEP47 | -0.166 |
| CEP23-CEP47 | -0.155 |
| CEP24-CEP47 | -0.144 |
| CEP25-CEP47 | -0.071 |
| CEP26-CEP47 | -0.155 |
| CEP27-CEP47 | -0.054 |
| CEP28-CEP47 | -0.036 |
| CEP29-CEP47 | -0.027 |
| CEP30-CEP47 | -0.133 |
| CEP31-CEP47 | -0.128 |
| CEP32-CEP47 | -0.128 |
| CEP33-CEP47 | -0.133 |
| CEP34-CEP47 | -0.054 |
| CEP35-CEP47 | -0.054 |
| CEP36-CEP47 | -0.133 |
| CEP37-CEP47 | -0.144 |
| CEP38-CEP47 | 0.063 |
| CEP39-CEP47 | -0.133 |
| CEP40-CEP47 | 0.107 |
| CEP41-CEP47 | 0.144 |
| CEP42-CEP47 | 0.069 |
| CEP43-CEP47 | 0.127 |
| CEP44-CEP47 | 0.304 |
| CEP45-CEP47 | 0.304 |
| CEP46-CEP47 | 0.246 |
| CEP1-CEP48 | -0.014 |
| CEP2-CEP48 | -0.130 |
| CEP3-CEP48 | -0.122 |
| CEP4-CEP48 | -0.079 |
| CEP5-CEP48 | -0.111 |
| CEP6-CEP48 | -0.062 |
| CEP7-CEP48 | -0.084 |
| CEP8-CEP48 | -0.141 |
| CEP9-CEP48 | -0.137 |
| CEP10-CEP48 | -0.060 |
| CEP11-CEP48 | -0.056 |
| CEP12-CEP48 | -0.142 |
| CEP13-CEP48 | -0.137 |
| CEP14-CEP48 | -0.097 |
| CEP15-CEP48 | -0.111 |
| CEP16-CEP48 | -0.100 |
| CEP17-CEP48 | -0.097 |
| CEP18-CEP48 | -0.083 |
| CEP19-CEP48 | -0.091 |
| CEP20-CEP48 | -0.141 |
| CEP21-CEP48 | -0.118 |
| CEP22-CEP48 | -0.114 |
| CEP23-CEP48 | -0.110 |
| CEP24-CEP48 | -0.130 |
| CEP25-CEP48 | -0.037 |
| CEP26-CEP48 | -0.110 |
| CEP27-CEP48 | -0.099 |
| CEP28-CEP48 | -0.119 |
| CEP29-CEP48 | -0.065 |
| CEP30-CEP48 | -0.126 |
| CEP31-CEP48 | -0.095 |
| CEP32-CEP48 | -0.076 |
| CEP33-CEP48 | -0.119 |
| CEP34-CEP48 | -0.130 |
| CEP35-CEP48 | -0.130 |
| CEP36-CEP48 | -0.119 |
| CEP37-CEP48 | -0.130 |
| CEP38-CEP48 | -0.103 |
| CEP39-CEP48 | -0.119 |
| CEP40-CEP48 | 0.200 |
| CEP41-CEP48 | 0.115 |
| CEP42-CEP48 | 0.103 |
| CEP43-CEP48 | 0.117 |
| CEP44-CEP48 | 0.102 |
| CEP45-CEP48 | 0.102 |
| CEP46-CEP48 | 0.120 |
| CEP47-CEP48 | 0.160 |
| CEP1-CEP49 | -0.130 |
| CEP2-CEP49 | -0.088 |
| CEP3-CEP49 | -0.028 |
| CEP4-CEP49 | -0.122 |
| CEP5-CEP49 | -0.122 |
| CEP6-CEP49 | -0.141 |
| CEP7-CEP49 | -0.163 |
| CEP8-CEP49 | -0.152 |
| CEP9-CEP49 | 0.000 |
| CEP10-CEP49 | -0.071 |
| CEP11-CEP49 | -0.099 |
| CEP12-CEP49 | -0.153 |
| CEP13-CEP49 | -0.148 |
| CEP14-CEP49 | -0.097 |
| CEP15-CEP49 | -0.122 |
| CEP16-CEP49 | -0.111 |
| CEP17-CEP49 | -0.097 |
| CEP18-CEP49 | -0.110 |
| CEP19-CEP49 | -0.133 |
| CEP20-CEP49 | -0.152 |
| CEP21-CEP49 | -0.086 |
| CEP22-CEP49 | -0.125 |
| CEP23-CEP49 | -0.026 |
| CEP24-CEP49 | -0.141 |
| CEP25-CEP49 | -0.101 |
| CEP26-CEP49 | -0.078 |
| CEP27-CEP49 | -0.110 |
| CEP28-CEP49 | -0.130 |
| CEP29-CEP49 | -0.092 |
| CEP30-CEP49 | -0.032 |
| CEP31-CEP49 | -0.079 |
| CEP32-CEP49 | -0.060 |
| CEP33-CEP49 | -0.130 |
| CEP34-CEP49 | -0.098 |
| CEP35-CEP49 | -0.098 |
| CEP36-CEP49 | -0.130 |
| CEP37-CEP49 | -0.141 |
| CEP38-CEP49 | -0.130 |
| CEP39-CEP49 | -0.130 |
| CEP40-CEP49 | 0.135 |
| CEP41-CEP49 | 0.024 |
| CEP42-CEP49 | 0.071 |
| CEP43-CEP49 | 0.052 |
| CEP44-CEP49 | 0.039 |
| CEP45-CEP49 | 0.039 |
| CEP46-CEP49 | 0.023 |
| CEP47-CEP49 | 0.011 |
| CEP48-CEP49 | 0.103 |
| CEP1-CEP50 | -0.141 |
| CEP2-CEP50 | -0.152 |
| CEP3-CEP50 | -0.111 |
| CEP4-CEP50 | -0.070 |
| CEP5-CEP50 | -0.133 |
| CEP6-CEP50 | -0.120 |
| CEP7-CEP50 | -0.077 |
| CEP8-CEP50 | -0.130 |
| CEP9-CEP50 | -0.153 |
| CEP10-CEP50 | -0.082 |
| CEP11-CEP50 | -0.047 |
| CEP12-CEP50 | -0.164 |
| CEP13-CEP50 | -0.159 |
| CEP14-CEP50 | -0.082 |
| CEP15-CEP50 | -0.133 |
| CEP16-CEP50 | -0.122 |
| CEP17-CEP50 | -0.082 |
| CEP18-CEP50 | -0.089 |
| CEP19-CEP50 | -0.048 |
| CEP20-CEP50 | -0.130 |
| CEP21-CEP50 | -0.134 |
| CEP22-CEP50 | -0.070 |
| CEP23-CEP50 | -0.126 |
| CEP24-CEP50 | -0.152 |
| CEP25-CEP50 | -0.112 |
| CEP26-CEP50 | -0.126 |
| CEP27-CEP50 | -0.088 |
| CEP28-CEP50 | -0.141 |
| CEP29-CEP50 | -0.071 |
| CEP30-CEP50 | -0.148 |
| CEP31-CEP50 | -0.096 |
| CEP32-CEP50 | -0.077 |
| CEP33-CEP50 | -0.141 |
| CEP34-CEP50 | -0.146 |
| CEP35-CEP50 | -0.146 |
| CEP36-CEP50 | -0.141 |
| CEP37-CEP50 | -0.119 |
| CEP38-CEP50 | -0.109 |
| CEP39-CEP50 | -0.141 |
| CEP40-CEP50 | 0.225 |
| CEP41-CEP50 | 0.429 |
| CEP42-CEP50 | 0.093 |
| CEP43-CEP50 | 0.208 |
| CEP44-CEP50 | 0.094 |
| CEP45-CEP50 | 0.094 |
| CEP46-CEP50 | 0.114 |
| CEP47-CEP50 | 0.101 |
| CEP48-CEP50 | 0.194 |
| CEP49-CEP50 | 0.118 |
| CEP1-CEP51 | -0.119 |
| CEP2-CEP51 | -0.103 |
| CEP3-CEP51 | -0.141 |
| CEP4-CEP51 | -0.130 |
| CEP5-CEP51 | -0.130 |
| CEP6-CEP51 | -0.130 |
| CEP7-CEP51 | -0.152 |
| CEP8-CEP51 | -0.088 |
| CEP9-CEP51 | -0.137 |
| CEP10-CEP51 | -0.044 |
| CEP11-CEP51 | -0.126 |
| CEP12-CEP51 | -0.142 |
| CEP13-CEP51 | -0.083 |
| CEP14-CEP51 | -0.148 |
| CEP15-CEP51 | -0.076 |
| CEP16-CEP51 | -0.119 |
| CEP17-CEP51 | -0.148 |
| CEP18-CEP51 | -0.137 |
| CEP19-CEP51 | -0.141 |
| CEP20-CEP51 | 0.009 |
| CEP21-CEP51 | -0.083 |
| CEP22-CEP51 | -0.099 |
| CEP23-CEP51 | -0.148 |
| CEP24-CEP51 | -0.130 |
| CEP25-CEP51 | -0.082 |
| CEP26-CEP51 | -0.148 |
| CEP27-CEP51 | -0.137 |
| CEP28-CEP51 | -0.022 |
| CEP29-CEP51 | -0.092 |
| CEP30-CEP51 | -0.126 |
| CEP31-CEP51 | -0.103 |
| CEP32-CEP51 | -0.103 |
| CEP33-CEP51 | -0.119 |
| CEP34-CEP51 | -0.076 |
| CEP35-CEP51 | -0.130 |
| CEP36-CEP51 | -0.065 |
| CEP37-CEP51 | -0.130 |
| CEP38-CEP51 | -0.092 |
| CEP39-CEP51 | -0.065 |
| CEP40-CEP51 | 0.092 |
| CEP41-CEP51 | 0.098 |
| CEP42-CEP51 | 0.202 |
| CEP43-CEP51 | 0.112 |
| CEP44-CEP51 | 0.171 |
| CEP45-CEP51 | 0.171 |
| CEP46-CEP51 | 0.200 |
| CEP47-CEP51 | 0.257 |
| CEP48-CEP51 | 0.034 |
| CEP49-CEP51 | 0.055 |
| CEP50-CEP51 | 0.044 |
| CEP1-CEP52 | -0.133 |
| CEP2-CEP52 | -0.078 |
| CEP3-CEP52 | -0.050 |
| CEP4-CEP52 | -0.144 |
| CEP5-CEP52 | -0.144 |
| CEP6-CEP52 | -0.144 |
| CEP7-CEP52 | -0.166 |
| CEP8-CEP52 | -0.128 |
| CEP9-CEP52 | -0.039 |
| CEP10-CEP52 | -0.085 |
| CEP11-CEP52 | -0.133 |
| CEP12-CEP52 | -0.156 |
| CEP13-CEP52 | -0.117 |
| CEP14-CEP52 | -0.155 |
| CEP15-CEP52 | -0.117 |
| CEP16-CEP52 | -0.133 |
| CEP17-CEP52 | -0.155 |
| CEP18-CEP52 | -0.144 |
| CEP19-CEP52 | -0.155 |
| CEP20-CEP52 | -0.032 |
| CEP21-CEP52 | -0.117 |
| CEP22-CEP52 | -0.140 |
| CEP23-CEP52 | -0.102 |
| CEP24-CEP52 | -0.144 |
| CEP25-CEP52 | -0.110 |
| CEP26-CEP52 | -0.155 |
| CEP27-CEP52 | -0.144 |
| CEP28-CEP52 | -0.036 |
| CEP29-CEP52 | -0.120 |
| CEP30-CEP52 | -0.028 |
| CEP31-CEP52 | -0.130 |
| CEP32-CEP52 | -0.130 |
| CEP33-CEP52 | -0.133 |
| CEP34-CEP52 | -0.117 |
| CEP35-CEP52 | -0.144 |
| CEP36-CEP52 | -0.106 |
| CEP37-CEP52 | -0.144 |
| CEP38-CEP52 | -0.120 |
| CEP39-CEP52 | -0.106 |
| CEP40-CEP52 | 0.202 |
| CEP41-CEP52 | 0.123 |
| CEP42-CEP52 | 0.043 |
| CEP43-CEP52 | 0.138 |
| CEP44-CEP52 | 0.036 |
| CEP45-CEP52 | 0.036 |
| CEP46-CEP52 | 0.225 |
| CEP47-CEP52 | 0.321 |
| CEP48-CEP52 | 0.157 |
| CEP49-CEP52 | 0.160 |
| CEP50-CEP52 | 0.167 |
| CEP51-CEP52 | 0.297 |
| CEP1-CEP53 | -0.130 |
| CEP2-CEP53 | -0.141 |
| CEP3-CEP53 | -0.100 |
| CEP4-CEP53 | -0.059 |
| CEP5-CEP53 | -0.122 |
| CEP6-CEP53 | -0.110 |
| CEP7-CEP53 | -0.066 |
| CEP8-CEP53 | -0.119 |
| CEP9-CEP53 | -0.148 |
| CEP10-CEP53 | -0.071 |
| CEP11-CEP53 | -0.036 |
| CEP12-CEP53 | -0.153 |
| CEP13-CEP53 | -0.148 |
| CEP14-CEP53 | -0.077 |
| CEP15-CEP53 | -0.122 |
| CEP16-CEP53 | -0.111 |
| CEP17-CEP53 | -0.077 |
| CEP18-CEP53 | -0.079 |
| CEP19-CEP53 | -0.037 |
| CEP20-CEP53 | -0.119 |
| CEP21-CEP53 | -0.129 |
| CEP22-CEP53 | -0.059 |
| CEP23-CEP53 | -0.121 |
| CEP24-CEP53 | -0.141 |
| CEP25-CEP53 | -0.101 |
| CEP26-CEP53 | -0.121 |
| CEP27-CEP53 | -0.077 |
| CEP28-CEP53 | -0.130 |
| CEP29-CEP53 | -0.060 |
| CEP30-CEP53 | -0.137 |
| CEP31-CEP53 | -0.090 |
| CEP32-CEP53 | -0.071 |
| CEP33-CEP53 | -0.130 |
| CEP34-CEP53 | -0.141 |
| CEP35-CEP53 | -0.141 |
| CEP36-CEP53 | -0.130 |
| CEP37-CEP53 | -0.108 |
| CEP38-CEP53 | -0.099 |
| CEP39-CEP53 | -0.130 |
| CEP40-CEP53 | 0.166 |
| CEP41-CEP53 | 0.049 |
| CEP42-CEP53 | 0.103 |
| CEP43-CEP53 | 0.149 |
| CEP44-CEP53 | 0.105 |
| CEP45-CEP53 | 0.105 |
| CEP46-CEP53 | 0.054 |
| CEP47-CEP53 | 0.042 |
| CEP48-CEP53 | 0.135 |
| CEP49-CEP53 | 0.124 |
| CEP50-CEP53 | 0.209 |
| CEP51-CEP53 | 0.054 |
| CEP52-CEP53 | 0.107 |
| CEP1-CEP54 | -0.144 |
| CEP2-CEP54 | -0.141 |
| CEP3-CEP54 | -0.147 |
| CEP4-CEP54 | -0.104 |
| CEP5-CEP54 | -0.136 |
| CEP6-CEP54 | -0.139 |
| CEP7-CEP54 | -0.161 |
| CEP8-CEP54 | -0.139 |
| CEP9-CEP54 | -0.112 |
| CEP10-CEP54 | -0.058 |
| CEP11-CEP54 | -0.074 |
| CEP12-CEP54 | -0.167 |
| CEP13-CEP54 | -0.128 |
| CEP14-CEP54 | -0.073 |
| CEP15-CEP54 | -0.109 |
| CEP16-CEP54 | -0.125 |
| CEP17-CEP54 | -0.073 |
| CEP18-CEP54 | -0.101 |
| CEP19-CEP54 | -0.115 |
| CEP20-CEP54 | -0.139 |
| CEP21-CEP54 | -0.066 |
| CEP22-CEP54 | -0.112 |
| CEP23-CEP54 | -0.085 |
| CEP24-CEP54 | -0.155 |
| CEP25-CEP54 | -0.101 |
| CEP26-CEP54 | -0.085 |
| CEP27-CEP54 | -0.117 |
| CEP28-CEP54 | -0.144 |
| CEP29-CEP54 | -0.076 |
| CEP30-CEP54 | -0.144 |
| CEP31-CEP54 | -0.064 |
| CEP32-CEP54 | -0.045 |
| CEP33-CEP54 | -0.144 |
| CEP34-CEP54 | -0.085 |
| CEP35-CEP54 | -0.112 |
| CEP36-CEP54 | -0.117 |
| CEP37-CEP54 | -0.155 |
| CEP38-CEP54 | -0.115 |
| CEP39-CEP54 | -0.117 |
| CEP40-CEP54 | 0.175 |
| CEP41-CEP54 | 0.106 |
| CEP42-CEP54 | 0.112 |
| CEP43-CEP54 | 0.134 |
| CEP44-CEP54 | 0.106 |
| CEP45-CEP54 | 0.106 |
| CEP46-CEP54 | 0.105 |
| CEP47-CEP54 | 0.105 |
| CEP48-CEP54 | 0.130 |
| CEP49-CEP54 | 0.178 |
| CEP50-CEP54 | 0.161 |
| CEP51-CEP54 | 0.135 |
| CEP52-CEP54 | 0.157 |
| CEP53-CEP54 | 0.166 |
| CEP1-CEP55 | -0.133 |
| CEP2-CEP55 | -0.092 |
| CEP3-CEP55 | -0.050 |
| CEP4-CEP55 | -0.144 |
| CEP5-CEP55 | -0.144 |
| CEP6-CEP55 | -0.144 |
| CEP7-CEP55 | -0.166 |
| CEP8-CEP55 | -0.155 |
| CEP9-CEP55 | -0.039 |
| CEP10-CEP55 | -0.112 |
| CEP11-CEP55 | -0.133 |
| CEP12-CEP55 | -0.156 |
| CEP13-CEP55 | -0.144 |
| CEP14-CEP55 | -0.155 |
| CEP15-CEP55 | -0.144 |
| CEP16-CEP55 | 0.119 |
| CEP17-CEP55 | -0.100 |
| CEP18-CEP55 | -0.089 |
| CEP19-CEP55 | -0.155 |
| CEP20-CEP55 | -0.155 |
| CEP21-CEP55 | -0.089 |
| CEP22-CEP55 | -0.112 |
| CEP23-CEP55 | -0.048 |
| CEP24-CEP55 | -0.144 |
| CEP25-CEP55 | -0.123 |
| CEP26-CEP55 | -0.155 |
| CEP27-CEP55 | -0.117 |
| CEP28-CEP55 | -0.133 |
| CEP29-CEP55 | -0.106 |
| CEP30-CEP55 | -0.028 |
| CEP31-CEP55 | -0.116 |
| CEP32-CEP55 | -0.116 |
| CEP33-CEP55 | -0.133 |
| CEP34-CEP55 | -0.116 |
| CEP35-CEP55 | -0.116 |
| CEP36-CEP55 | -0.106 |
| CEP37-CEP55 | -0.117 |
| CEP38-CEP55 | -0.133 |
| CEP39-CEP55 | -0.078 |
| CEP40-CEP55 | 0.053 |
| CEP41-CEP55 | 0.017 |
| CEP42-CEP55 | 0.096 |
| CEP43-CEP55 | 0.031 |
| CEP44-CEP55 | 0.089 |
| CEP45-CEP55 | 0.089 |
| CEP46-CEP55 | 0.022 |
| CEP47-CEP55 | 0.046 |
| CEP48-CEP55 | 0.022 |
| CEP49-CEP55 | 0.567 |
| CEP50-CEP55 | 0.031 |
| CEP51-CEP55 | 0.143 |
| CEP52-CEP55 | 0.142 |
| CEP53-CEP55 | 0.042 |
| CEP54-CEP55 | 0.078 |
| CEP1-CEP56 | -0.144 |
| CEP2-CEP56 | -0.128 |
| CEP3-CEP56 | -0.147 |
| CEP4-CEP56 | -0.136 |
| CEP5-CEP56 | -0.136 |
| CEP6-CEP56 | -0.155 |
| CEP7-CEP56 | -0.177 |
| CEP8-CEP56 | -0.112 |
| CEP9-CEP56 | -0.112 |
| CEP10-CEP56 | -0.031 |
| CEP11-CEP56 | -0.106 |
| CEP12-CEP56 | -0.167 |
| CEP13-CEP56 | -0.101 |
| CEP14-CEP56 | -0.104 |
| CEP15-CEP56 | -0.082 |
| CEP16-CEP56 | -0.125 |
| CEP17-CEP56 | -0.104 |
| CEP18-CEP56 | -0.117 |
| CEP19-CEP56 | -0.147 |
| CEP20-CEP56 | -0.112 |
| CEP21-CEP56 | -0.039 |
| CEP22-CEP56 | -0.085 |
| CEP23-CEP56 | -0.085 |
| CEP24-CEP56 | -0.155 |
| CEP25-CEP56 | -0.088 |
| CEP26-CEP56 | -0.085 |
| CEP27-CEP56 | -0.117 |
| CEP28-CEP56 | -0.144 |
| CEP29-CEP56 | -0.079 |
| CEP30-CEP56 | -0.144 |
| CEP31-CEP56 | -0.066 |
| CEP32-CEP56 | -0.047 |
| CEP33-CEP56 | -0.144 |
| CEP34-CEP56 | -0.059 |
| CEP35-CEP56 | -0.112 |
| CEP36-CEP56 | -0.090 |
| CEP37-CEP56 | -0.155 |
| CEP38-CEP56 | -0.117 |
| CEP39-CEP56 | -0.090 |
| CEP40-CEP56 | 0.173 |
| CEP41-CEP56 | 0.061 |
| CEP42-CEP56 | 0.084 |
| CEP43-CEP56 | 0.089 |
| CEP44-CEP56 | 0.077 |
| CEP45-CEP56 | 0.077 |
| CEP46-CEP56 | 0.061 |
| CEP47-CEP56 | 0.047 |
| CEP48-CEP56 | 0.114 |
| CEP49-CEP56 | 0.335 |
| CEP50-CEP56 | 0.129 |
| CEP51-CEP56 | 0.119 |
| CEP52-CEP56 | 0.171 |
| CEP53-CEP56 | 0.135 |
| CEP54-CEP56 | 0.240 |
| CEP55-CEP56 | 0.236 |
| CEP1-CEP57 | -0.144 |
| CEP2-CEP57 | -0.150 |
| CEP3-CEP57 | -0.142 |
| CEP4-CEP57 | -0.099 |
| CEP5-CEP57 | -0.130 |
| CEP6-CEP57 | -0.134 |
| CEP7-CEP57 | -0.156 |
| CEP8-CEP57 | -0.161 |
| CEP9-CEP57 | -0.155 |
| CEP10-CEP57 | -0.085 |
| CEP11-CEP57 | -0.074 |
| CEP12-CEP57 | -0.162 |
| CEP13-CEP57 | -0.150 |
| CEP14-CEP57 | -0.110 |
| CEP15-CEP57 | -0.130 |
| CEP16-CEP57 | -0.125 |
| CEP17-CEP57 | -0.110 |
| CEP18-CEP57 | 0.219 |
| CEP19-CEP57 | -0.110 |
| CEP20-CEP57 | -0.161 |
| CEP21-CEP57 | -0.136 |
| CEP22-CEP57 | -0.134 |
| CEP23-CEP57 | -0.122 |
| CEP24-CEP57 | -0.150 |
| CEP25-CEP57 | -0.109 |
| CEP26-CEP57 | -0.122 |
| CEP27-CEP57 | -0.117 |
| CEP28-CEP57 | -0.144 |
| CEP29-CEP57 | -0.090 |
| CEP30-CEP57 | -0.144 |
| CEP31-CEP57 | -0.120 |
| CEP32-CEP57 | -0.101 |
| CEP33-CEP57 | -0.144 |
| CEP34-CEP57 | -0.155 |
| CEP35-CEP57 | -0.155 |
| CEP36-CEP57 | -0.144 |
| CEP37-CEP57 | -0.155 |
| CEP38-CEP57 | -0.128 |
| CEP39-CEP57 | -0.144 |
| CEP40-CEP57 | 0.119 |
| CEP41-CEP57 | 0.087 |
| CEP42-CEP57 | 0.099 |
| CEP43-CEP57 | 0.120 |
| CEP44-CEP57 | 0.092 |
| CEP45-CEP57 | 0.092 |
| CEP46-CEP57 | 0.092 |
| CEP47-CEP57 | 0.104 |
| CEP48-CEP57 | 0.088 |
| CEP49-CEP57 | 0.093 |
| CEP50-CEP57 | 0.113 |
| CEP51-CEP57 | 0.107 |
| CEP52-CEP57 | 0.101 |
| CEP53-CEP57 | 0.214 |
| CEP54-CEP57 | 0.187 |
| CEP55-CEP57 | 0.078 |
| CEP56-CEP57 | 0.129 |
| CEP1-CEP58 | -0.127 |
| CEP2-CEP58 | -0.133 |
| CEP3-CEP58 | -0.092 |
| CEP4-CEP58 | -0.051 |
| CEP5-CEP58 | -0.114 |
| CEP6-CEP58 | -0.101 |
| CEP7-CEP58 | -0.058 |
| CEP8-CEP58 | -0.111 |
| CEP9-CEP58 | -0.152 |
| CEP10-CEP58 | -0.068 |
| CEP11-CEP58 | -0.040 |
| CEP12-CEP58 | -0.145 |
| CEP13-CEP58 | -0.147 |
| CEP14-CEP58 | -0.075 |
| CEP15-CEP58 | -0.114 |
| CEP16-CEP58 | -0.108 |
| CEP17-CEP58 | -0.075 |
| CEP18-CEP58 | -0.077 |
| CEP19-CEP58 | -0.029 |
| CEP20-CEP58 | -0.111 |
| CEP21-CEP58 | -0.133 |
| CEP22-CEP58 | -0.051 |
| CEP23-CEP58 | -0.119 |
| CEP24-CEP58 | -0.133 |
| CEP25-CEP58 | -0.093 |
| CEP26-CEP58 | -0.119 |
| CEP27-CEP58 | -0.081 |
| CEP28-CEP58 | -0.127 |
| CEP29-CEP58 | -0.058 |
| CEP30-CEP58 | -0.141 |
| CEP31-CEP58 | -0.087 |
| CEP32-CEP58 | -0.068 |
| CEP33-CEP58 | -0.127 |
| CEP34-CEP58 | -0.138 |
| CEP35-CEP58 | -0.138 |
| CEP36-CEP58 | -0.127 |
| CEP37-CEP58 | -0.105 |
| CEP38-CEP58 | -0.096 |
| CEP39-CEP58 | -0.127 |
| CEP40-CEP58 | 0.144 |
| CEP41-CEP58 | 0.027 |
| CEP42-CEP58 | 0.106 |
| CEP43-CEP58 | 0.127 |
| CEP44-CEP58 | 0.083 |
| CEP45-CEP58 | 0.083 |
| CEP46-CEP58 | 0.032 |
| CEP47-CEP58 | -0.005 |
| CEP48-CEP58 | 0.113 |
| CEP49-CEP58 | 0.102 |
| CEP50-CEP58 | 0.187 |
| CEP51-CEP58 | 0.032 |
| CEP52-CEP58 | 0.060 |
| CEP53-CEP58 | 0.287 |
| CEP54-CEP58 | 0.119 |
| CEP55-CEP58 | -0.005 |
| CEP56-CEP58 | 0.088 |
| CEP57-CEP58 | 0.487 |
| CEP1-CEP59 | -0.133 |
| CEP2-CEP59 | -0.131 |
| CEP3-CEP59 | -0.155 |
| CEP4-CEP59 | -0.113 |
| CEP5-CEP59 | -0.144 |
| CEP6-CEP59 | -0.128 |
| CEP7-CEP59 | -0.151 |
| CEP8-CEP59 | -0.128 |
| CEP9-CEP59 | -0.144 |
| CEP10-CEP59 | -0.085 |
| CEP11-CEP59 | -0.101 |
| CEP12-CEP59 | -0.156 |
| CEP13-CEP59 | -0.117 |
| CEP14-CEP59 | -0.123 |
| CEP15-CEP59 | -0.117 |
| CEP16-CEP59 | 0.119 |
| CEP17-CEP59 | -0.123 |
| CEP18-CEP59 | -0.128 |
| CEP19-CEP59 | -0.124 |
| CEP20-CEP59 | -0.128 |
| CEP21-CEP59 | -0.117 |
| CEP22-CEP59 | -0.140 |
| CEP23-CEP59 | -0.155 |
| CEP24-CEP59 | -0.144 |
| CEP25-CEP59 | -0.110 |
| CEP26-CEP59 | -0.155 |
| CEP27-CEP59 | -0.144 |
| CEP28-CEP59 | -0.133 |
| CEP29-CEP59 | -0.104 |
| CEP30-CEP59 | -0.133 |
| CEP31-CEP59 | -0.115 |
| CEP32-CEP59 | -0.115 |
| CEP33-CEP59 | -0.133 |
| CEP34-CEP59 | -0.117 |
| CEP35-CEP59 | -0.144 |
| CEP36-CEP59 | -0.106 |
| CEP37-CEP59 | -0.144 |
| CEP38-CEP59 | -0.104 |
| CEP39-CEP59 | -0.106 |
| CEP40-CEP59 | 0.106 |
| CEP41-CEP59 | 0.111 |
| CEP42-CEP59 | 0.085 |
| CEP43-CEP59 | 0.125 |
| CEP44-CEP59 | 0.078 |
| CEP45-CEP59 | 0.078 |
| CEP46-CEP59 | 0.116 |
| CEP47-CEP59 | 0.115 |
| CEP48-CEP59 | 0.061 |
| CEP49-CEP59 | 0.066 |
| CEP50-CEP59 | 0.086 |
| CEP51-CEP59 | 0.145 |
| CEP52-CEP59 | 0.126 |
| CEP53-CEP59 | 0.187 |
| CEP54-CEP59 | 0.173 |
| CEP55-CEP59 | 0.593 |
| CEP56-CEP59 | 0.129 |
| CEP57-CEP59 | 0.249 |
| CEP58-CEP59 | 0.140 |
| CEP1-CEP60 | -0.130 |
| CEP2-CEP60 | -0.127 |
| CEP3-CEP60 | -0.133 |
| CEP4-CEP60 | -0.090 |
| CEP5-CEP60 | -0.122 |
| CEP6-CEP60 | -0.125 |
| CEP7-CEP60 | -0.147 |
| CEP8-CEP60 | -0.125 |
| CEP9-CEP60 | -0.105 |
| CEP10-CEP60 | -0.044 |
| CEP11-CEP60 | -0.067 |
| CEP12-CEP60 | -0.153 |
| CEP13-CEP60 | -0.121 |
| CEP14-CEP60 | -0.066 |
| CEP15-CEP60 | -0.095 |
| CEP16-CEP60 | -0.111 |
| CEP17-CEP60 | -0.066 |
| CEP18-CEP60 | -0.094 |
| CEP19-CEP60 | -0.101 |
| CEP20-CEP60 | -0.125 |
| CEP21-CEP60 | -0.059 |
| CEP22-CEP60 | -0.098 |
| CEP23-CEP60 | -0.078 |
| CEP24-CEP60 | -0.141 |
| CEP25-CEP60 | -0.087 |
| CEP26-CEP60 | -0.078 |
| CEP27-CEP60 | -0.110 |
| CEP28-CEP60 | -0.130 |
| CEP29-CEP60 | -0.062 |
| CEP30-CEP60 | -0.137 |
| CEP31-CEP60 | -0.050 |
| CEP32-CEP60 | -0.031 |
| CEP33-CEP60 | -0.130 |
| CEP34-CEP60 | -0.071 |
| CEP35-CEP60 | -0.098 |
| CEP36-CEP60 | -0.103 |
| CEP37-CEP60 | -0.141 |
| CEP38-CEP60 | -0.101 |
| CEP39-CEP60 | -0.103 |
| CEP40-CEP60 | 0.164 |
| CEP41-CEP60 | 0.053 |
| CEP42-CEP60 | 0.084 |
| CEP43-CEP60 | 0.081 |
| CEP44-CEP60 | 0.053 |
| CEP45-CEP60 | 0.053 |
| CEP46-CEP60 | 0.052 |
| CEP47-CEP60 | 0.027 |
| CEP48-CEP60 | 0.119 |
| CEP49-CEP60 | 0.167 |
| CEP50-CEP60 | 0.150 |
| CEP51-CEP60 | 0.082 |
| CEP52-CEP60 | 0.121 |
| CEP53-CEP60 | 0.245 |
| CEP54-CEP60 | 0.207 |
| CEP55-CEP60 | 0.042 |
| CEP56-CEP60 | 0.204 |
| CEP57-CEP60 | 0.199 |
| CEP58-CEP60 | 0.223 |
| CEP59-CEP60 | 0.185 |
| CEP1-CEP61 | -0.141 |
| CEP2-CEP61 | -0.139 |
| CEP3-CEP61 | -0.078 |
| CEP4-CEP61 | -0.102 |
| CEP5-CEP61 | -0.133 |
| CEP6-CEP61 | -0.137 |
| CEP7-CEP61 | -0.027 |
| CEP8-CEP61 | -0.071 |
| CEP9-CEP61 | -0.159 |
| CEP10-CEP61 | -0.055 |
| CEP11-CEP61 | -0.079 |
| CEP12-CEP61 | -0.164 |
| CEP13-CEP61 | -0.133 |
| CEP14-CEP61 | -0.120 |
| CEP15-CEP61 | -0.106 |
| CEP16-CEP61 | -0.122 |
| CEP17-CEP61 | -0.120 |
| CEP18-CEP61 | -0.105 |
| CEP19-CEP61 | -0.047 |
| CEP20-CEP61 | -0.071 |
| CEP21-CEP61 | -0.113 |
| CEP22-CEP61 | 0.022 |
| CEP23-CEP61 | -0.132 |
| CEP24-CEP61 | -0.152 |
| CEP25-CEP61 | -0.099 |
| CEP26-CEP61 | -0.132 |
| CEP27-CEP61 | -0.055 |
| CEP28-CEP61 | -0.141 |
| CEP29-CEP61 | -0.074 |
| CEP30-CEP61 | -0.148 |
| CEP31-CEP61 | -0.104 |
| CEP32-CEP61 | -0.085 |
| CEP33-CEP61 | -0.141 |
| CEP34-CEP61 | -0.125 |
| CEP35-CEP61 | -0.152 |
| CEP36-CEP61 | -0.114 |
| CEP37-CEP61 | -0.087 |
| CEP38-CEP61 | -0.112 |
| CEP39-CEP61 | -0.114 |
| CEP40-CEP61 | 0.153 |
| CEP41-CEP61 | 0.078 |
| CEP42-CEP61 | 0.181 |
| CEP43-CEP61 | 0.243 |
| CEP44-CEP61 | 0.215 |
| CEP45-CEP61 | 0.215 |
| CEP46-CEP61 | 0.083 |
| CEP47-CEP61 | 0.057 |
| CEP48-CEP61 | 0.108 |
| CEP49-CEP61 | 0.113 |
| CEP50-CEP61 | 0.199 |
| CEP51-CEP61 | 0.112 |
| CEP52-CEP61 | 0.110 |
| CEP53-CEP61 | 0.210 |
| CEP54-CEP61 | 0.195 |
| CEP55-CEP61 | 0.031 |
| CEP56-CEP61 | 0.150 |
| CEP57-CEP61 | 0.139 |
| CEP58-CEP61 | 0.188 |
| CEP59-CEP61 | 0.125 |
| CEP60-CEP61 | 0.142 |
| CEP1-CEP62 | -0.130 |
| CEP2-CEP62 | -0.075 |
| CEP3-CEP62 | -0.028 |
| CEP4-CEP62 | -0.122 |
| CEP5-CEP62 | -0.122 |
| CEP6-CEP62 | -0.141 |
| CEP7-CEP62 | -0.163 |
| CEP8-CEP62 | -0.125 |
| CEP9-CEP62 | 0.000 |
| CEP10-CEP62 | -0.044 |
| CEP11-CEP62 | -0.099 |
| CEP12-CEP62 | -0.153 |
| CEP13-CEP62 | -0.121 |
| CEP14-CEP62 | -0.097 |
| CEP15-CEP62 | -0.095 |
| CEP16-CEP62 | -0.111 |
| CEP17-CEP62 | -0.042 |
| CEP18-CEP62 | -0.055 |
| CEP19-CEP62 | -0.133 |
| CEP20-CEP62 | -0.125 |
| CEP21-CEP62 | -0.004 |
| CEP22-CEP62 | -0.043 |
| CEP23-CEP62 | 0.029 |
| CEP24-CEP62 | -0.141 |
| CEP25-CEP62 | -0.087 |
| CEP26-CEP62 | -0.078 |
| CEP27-CEP62 | -0.082 |
| CEP28-CEP62 | -0.130 |
| CEP29-CEP62 | -0.051 |
| CEP30-CEP62 | -0.032 |
| CEP31-CEP62 | -0.038 |
| CEP32-CEP62 | -0.019 |
| CEP33-CEP62 | -0.130 |
| CEP34-CEP62 | -0.044 |
| CEP35-CEP62 | -0.071 |
| CEP36-CEP62 | -0.076 |
| CEP37-CEP62 | -0.114 |
| CEP38-CEP62 | -0.116 |
| CEP39-CEP62 | -0.048 |
| CEP40-CEP62 | 0.125 |
| CEP41-CEP62 | 0.014 |
| CEP42-CEP62 | 0.151 |
| CEP43-CEP62 | 0.042 |
| CEP44-CEP62 | 0.119 |
| CEP45-CEP62 | 0.119 |
| CEP46-CEP62 | 0.013 |
| CEP47-CEP62 | -0.001 |
| CEP48-CEP62 | 0.080 |
| CEP49-CEP62 | 0.196 |
| CEP50-CEP62 | 0.095 |
| CEP51-CEP62 | 0.148 |
| CEP52-CEP62 | 0.150 |
| CEP53-CEP62 | 0.100 |
| CEP54-CEP62 | 0.168 |
| CEP55-CEP62 | 0.201 |
| CEP56-CEP62 | 0.181 |
| CEP57-CEP62 | 0.070 |
| CEP58-CEP62 | 0.078 |
| CEP59-CEP62 | 0.056 |
| CEP60-CEP62 | 0.157 |
| CEP61-CEP62 | 0.103 |
| CEP1-CEP63 | -0.003 |
| CEP2-CEP63 | -0.119 |
| CEP3-CEP63 | -0.130 |
| CEP4-CEP63 | -0.087 |
| CEP5-CEP63 | -0.119 |
| CEP6-CEP63 | -0.050 |
| CEP7-CEP63 | -0.073 |
| CEP8-CEP63 | -0.130 |
| CEP9-CEP63 | -0.125 |
| CEP10-CEP63 | -0.087 |
| CEP11-CEP63 | -0.083 |
| CEP12-CEP63 | -0.131 |
| CEP13-CEP63 | -0.126 |
| CEP14-CEP63 | -0.105 |
| CEP15-CEP63 | -0.119 |
| CEP16-CEP63 | -0.108 |
| CEP17-CEP63 | -0.105 |
| CEP18-CEP63 | -0.110 |
| CEP19-CEP63 | -0.098 |
| CEP20-CEP63 | -0.033 |
| CEP21-CEP63 | -0.125 |
| CEP22-CEP63 | -0.141 |
| CEP23-CEP63 | -0.136 |
| CEP24-CEP63 | -0.119 |
| CEP25-CEP63 | -0.045 |
| CEP26-CEP63 | -0.136 |
| CEP27-CEP63 | -0.126 |
| CEP28-CEP63 | -0.011 |
| CEP29-CEP63 | -0.092 |
| CEP30-CEP63 | -0.115 |
| CEP31-CEP63 | -0.103 |
| CEP32-CEP63 | -0.103 |
| CEP33-CEP63 | -0.108 |
| CEP34-CEP63 | -0.118 |
| CEP35-CEP63 | -0.118 |
| CEP36-CEP63 | -0.108 |
| CEP37-CEP63 | -0.119 |
| CEP38-CEP63 | -0.092 |
| CEP39-CEP63 | -0.108 |
| CEP40-CEP63 | 0.125 |
| CEP41-CEP63 | 0.168 |
| CEP42-CEP63 | 0.112 |
| CEP43-CEP63 | 0.145 |
| CEP44-CEP63 | 0.112 |
| CEP45-CEP63 | 0.112 |
| CEP46-CEP63 | 0.264 |
| CEP47-CEP63 | 0.401 |
| CEP48-CEP63 | 0.178 |
| CEP49-CEP63 | 0.029 |
| CEP50-CEP63 | 0.119 |
| CEP51-CEP63 | 0.275 |
| CEP52-CEP63 | 0.314 |
| CEP53-CEP63 | 0.060 |
| CEP54-CEP63 | 0.098 |
| CEP55-CEP63 | 0.027 |
| CEP56-CEP63 | 0.040 |
| CEP57-CEP63 | 0.097 |
| CEP58-CEP63 | 0.038 |
| CEP59-CEP63 | 0.108 |
| CEP60-CEP63 | 0.045 |
| CEP61-CEP63 | 0.075 |
| CEP62-CEP63 | 0.006 |
| CEP1-CEP64 | -0.003 |
| CEP2-CEP64 | -0.105 |
| CEP3-CEP64 | -0.111 |
| CEP4-CEP64 | -0.100 |
| CEP5-CEP64 | -0.100 |
| CEP6-CEP64 | -0.066 |
| CEP7-CEP64 | -0.089 |
| CEP8-CEP64 | -0.103 |
| CEP9-CEP64 | -0.125 |
| CEP10-CEP64 | -0.022 |
| CEP11-CEP64 | -0.076 |
| CEP12-CEP64 | -0.131 |
| CEP13-CEP64 | -0.099 |
| CEP14-CEP64 | -0.117 |
| CEP15-CEP64 | -0.073 |
| CEP16-CEP64 | -0.089 |
| CEP17-CEP64 | -0.117 |
| CEP18-CEP64 | -0.088 |
| CEP19-CEP64 | -0.111 |
| CEP20-CEP64 | -0.103 |
| CEP21-CEP64 | -0.080 |
| CEP22-CEP64 | -0.076 |
| CEP23-CEP64 | -0.098 |
| CEP24-CEP64 | -0.119 |
| CEP25-CEP64 | -0.013 |
| CEP26-CEP64 | -0.098 |
| CEP27-CEP64 | -0.088 |
| CEP28-CEP64 | -0.108 |
| CEP29-CEP64 | -0.056 |
| CEP30-CEP64 | -0.115 |
| CEP31-CEP64 | -0.086 |
| CEP32-CEP64 | -0.067 |
| CEP33-CEP64 | -0.108 |
| CEP34-CEP64 | -0.092 |
| CEP35-CEP64 | -0.118 |
| CEP36-CEP64 | -0.081 |
| CEP37-CEP64 | -0.119 |
| CEP38-CEP64 | -0.094 |
| CEP39-CEP64 | -0.081 |
| CEP40-CEP64 | 0.091 |
| CEP41-CEP64 | 0.096 |
| CEP42-CEP64 | 0.254 |
| CEP43-CEP64 | 0.092 |
| CEP44-CEP64 | 0.253 |
| CEP45-CEP64 | 0.253 |
| CEP46-CEP64 | 0.095 |
| CEP47-CEP64 | 0.122 |
| CEP48-CEP64 | 0.130 |
| CEP49-CEP64 | 0.067 |
| CEP50-CEP64 | 0.056 |
| CEP51-CEP64 | 0.198 |
| CEP52-CEP64 | 0.064 |
| CEP53-CEP64 | 0.067 |
| CEP54-CEP64 | 0.134 |
| CEP55-CEP64 | 0.117 |
| CEP56-CEP64 | 0.105 |
| CEP57-CEP64 | 0.120 |
| CEP58-CEP64 | 0.045 |
| CEP59-CEP64 | 0.106 |
| CEP60-CEP64 | 0.081 |
| CEP61-CEP64 | 0.111 |
| CEP62-CEP64 | 0.147 |
| CEP63-CEP64 | 0.146 |
| CEP1-CEP65 | -0.130 |
| CEP2-CEP65 | -0.121 |
| CEP3-CEP65 | -0.132 |
| CEP4-CEP65 | -0.058 |
| CEP5-CEP65 | -0.121 |
| CEP6-CEP65 | -0.070 |
| CEP7-CEP65 | -0.092 |
| CEP8-CEP65 | -0.113 |
| CEP9-CEP65 | -0.085 |
| CEP10-CEP65 | -0.109 |
| CEP11-CEP65 | -0.074 |
| CEP12-CEP65 | -0.113 |
| CEP13-CEP65 | -0.108 |
| CEP14-CEP65 | -0.033 |
| CEP15-CEP65 | -0.121 |
| CEP16-CEP65 | -0.130 |
| CEP17-CEP65 | 0.021 |
| CEP18-CEP65 | -0.062 |
| CEP19-CEP65 | -0.069 |
| CEP20-CEP65 | -0.152 |
| CEP21-CEP65 | -0.031 |
| CEP22-CEP65 | -0.108 |
| CEP23-CEP65 | -0.061 |
| CEP24-CEP65 | -0.101 |
| CEP25-CEP65 | -0.100 |
| CEP26-CEP65 | -0.116 |
| CEP27-CEP65 | -0.121 |
| CEP28-CEP65 | -0.130 |
| CEP29-CEP65 | -0.071 |
| CEP30-CEP65 | -0.117 |
| CEP31-CEP65 | -0.020 |
| CEP32-CEP65 | -0.039 |
| CEP33-CEP65 | -0.090 |
| CEP34-CEP65 | -0.071 |
| CEP35-CEP65 | -0.071 |
| CEP36-CEP65 | -0.102 |
| CEP37-CEP65 | -0.114 |
| CEP38-CEP65 | -0.098 |
| CEP39-CEP65 | -0.075 |
| CEP40-CEP65 | 0.105 |
| CEP41-CEP65 | 0.071 |
| CEP42-CEP65 | 0.009 |
| CEP43-CEP65 | 0.060 |
| CEP44-CEP65 | -0.022 |
| CEP45-CEP65 | -0.022 |
| CEP46-CEP65 | 0.051 |
| CEP47-CEP65 | 0.031 |
| CEP48-CEP65 | 0.073 |
| CEP49-CEP65 | 0.105 |
| CEP50-CEP65 | 0.120 |
| CEP51-CEP65 | 0.031 |
| CEP52-CEP65 | 0.084 |
| CEP53-CEP65 | 0.215 |
| CEP54-CEP65 | 0.148 |
| CEP55-CEP65 | 0.058 |
| CEP56-CEP65 | 0.116 |
| CEP57-CEP65 | 0.153 |
| CEP58-CEP65 | 0.193 |
| CEP59-CEP65 | 0.164 |
| CEP60-CEP65 | 0.227 |
| CEP61-CEP65 | 0.083 |
| CEP62-CEP65 | 0.121 |
| CEP63-CEP65 | 0.037 |
| CEP64-CEP65 | 0.006 |
| CEP1-CEP66 | -0.118 |
| CEP2-CEP66 | -0.129 |
| CEP3-CEP66 | -0.122 |
| CEP4-CEP66 | -0.047 |
| CEP5-CEP66 | -0.110 |
| CEP6-CEP66 | -0.098 |
| CEP7-CEP66 | -0.120 |
| CEP8-CEP66 | -0.141 |
| CEP9-CEP66 | -0.094 |
| CEP10-CEP66 | -0.059 |
| CEP11-CEP66 | -0.024 |
| CEP12-CEP66 | -0.141 |
| CEP13-CEP66 | -0.136 |
| CEP14-CEP66 | -0.023 |
| CEP15-CEP66 | -0.110 |
| CEP16-CEP66 | -0.099 |
| CEP17-CEP66 | -0.023 |
| CEP18-CEP66 | -0.067 |
| CEP19-CEP66 | -0.059 |
| CEP20-CEP66 | -0.141 |
| CEP21-CEP66 | -0.075 |
| CEP22-CEP66 | -0.114 |
| CEP23-CEP66 | -0.067 |
| CEP24-CEP66 | -0.129 |
| CEP25-CEP66 | -0.089 |
| CEP26-CEP66 | -0.067 |
| CEP27-CEP66 | -0.098 |
| CEP28-CEP66 | -0.118 |
| CEP29-CEP66 | -0.049 |
| CEP30-CEP66 | -0.125 |
| CEP31-CEP66 | -0.036 |
| CEP32-CEP66 | -0.017 |
| CEP33-CEP66 | -0.118 |
| CEP34-CEP66 | -0.087 |
| CEP35-CEP66 | -0.087 |
| CEP36-CEP66 | -0.118 |
| CEP37-CEP66 | -0.130 |
| CEP38-CEP66 | -0.087 |
| CEP39-CEP66 | -0.118 |
| CEP40-CEP66 | 0.141 |
| CEP41-CEP66 | 0.066 |
| CEP42-CEP66 | 0.045 |
| CEP43-CEP66 | 0.057 |
| CEP44-CEP66 | 0.045 |
| CEP45-CEP66 | 0.045 |
| CEP46-CEP66 | 0.060 |
| CEP47-CEP66 | 0.048 |
| CEP48-CEP66 | 0.141 |
| CEP49-CEP66 | 0.141 |
| CEP50-CEP66 | 0.156 |
| CEP51-CEP66 | 0.029 |
| CEP52-CEP66 | 0.082 |
| CEP53-CEP66 | 0.251 |
| CEP54-CEP66 | 0.183 |
| CEP55-CEP66 | 0.017 |
| CEP56-CEP66 | 0.152 |
| CEP57-CEP66 | 0.189 |
| CEP58-CEP66 | 0.229 |
| CEP59-CEP66 | 0.161 |
| CEP60-CEP66 | 0.262 |
| CEP61-CEP66 | 0.118 |
| CEP62-CEP66 | 0.117 |
| CEP63-CEP66 | 0.072 |
| CEP64-CEP66 | 0.078 |
| CEP65-CEP66 | 0.232 |
| CEP1-CEP67 | -0.130 |
| CEP2-CEP67 | -0.128 |
| CEP3-CEP67 | -0.100 |
| CEP4-CEP67 | -0.091 |
| CEP5-CEP67 | -0.122 |
| CEP6-CEP67 | -0.125 |
| CEP7-CEP67 | -0.082 |
| CEP8-CEP67 | -0.093 |
| CEP9-CEP67 | -0.148 |
| CEP10-CEP67 | -0.044 |
| CEP11-CEP67 | -0.067 |
| CEP12-CEP67 | -0.153 |
| CEP13-CEP67 | -0.121 |
| CEP14-CEP67 | -0.108 |
| CEP15-CEP67 | -0.095 |
| CEP16-CEP67 | -0.111 |
| CEP17-CEP67 | -0.108 |
| CEP18-CEP67 | -0.094 |
| CEP19-CEP67 | -0.069 |
| CEP20-CEP67 | -0.093 |
| CEP21-CEP67 | -0.102 |
| CEP22-CEP67 | -0.033 |
| CEP23-CEP67 | -0.121 |
| CEP24-CEP67 | -0.141 |
| CEP25-CEP67 | -0.088 |
| CEP26-CEP67 | -0.121 |
| CEP27-CEP67 | -0.077 |
| CEP28-CEP67 | -0.130 |
| CEP29-CEP67 | -0.063 |
| CEP30-CEP67 | -0.137 |
| CEP31-CEP67 | -0.093 |
| CEP32-CEP67 | -0.074 |
| CEP33-CEP67 | -0.130 |
| CEP34-CEP67 | -0.114 |
| CEP35-CEP67 | -0.141 |
| CEP36-CEP67 | -0.103 |
| CEP37-CEP67 | -0.108 |
| CEP38-CEP67 | -0.101 |
| CEP39-CEP67 | -0.103 |
| CEP40-CEP67 | 0.164 |
| CEP41-CEP67 | 0.047 |
| CEP42-CEP67 | 0.117 |
| CEP43-CEP67 | 0.146 |
| CEP44-CEP67 | 0.118 |
| CEP45-CEP67 | 0.118 |
| CEP46-CEP67 | 0.052 |
| CEP47-CEP67 | 0.026 |
| CEP48-CEP67 | 0.119 |
| CEP49-CEP67 | 0.281 |
| CEP50-CEP67 | 0.177 |
| CEP51-CEP67 | 0.081 |
| CEP52-CEP67 | 0.121 |
| CEP53-CEP67 | 0.188 |
| CEP54-CEP67 | 0.164 |
| CEP55-CEP67 | 0.200 |
| CEP56-CEP67 | 0.319 |
| CEP57-CEP67 | 0.108 |
| CEP58-CEP67 | 0.166 |
| CEP59-CEP67 | 0.095 |
| CEP60-CEP67 | 0.153 |
| CEP61-CEP67 | 0.207 |
| CEP62-CEP67 | 0.114 |
| CEP63-CEP67 | 0.044 |
| CEP64-CEP67 | 0.080 |
| CEP65-CEP67 | 0.094 |
| CEP66-CEP67 | 0.129 |
| CEP1-CEP68 | -0.133 |
| CEP2-CEP68 | -0.117 |
| CEP3-CEP68 | -0.155 |
| CEP4-CEP68 | -0.144 |
| CEP5-CEP68 | -0.144 |
| CEP6-CEP68 | -0.144 |
| CEP7-CEP68 | -0.166 |
| CEP8-CEP68 | -0.102 |
| CEP9-CEP68 | -0.144 |
| CEP10-CEP68 | -0.058 |
| CEP11-CEP68 | -0.133 |
| CEP12-CEP68 | -0.156 |
| CEP13-CEP68 | -0.090 |
| CEP14-CEP68 | -0.155 |
| CEP15-CEP68 | -0.090 |
| CEP16-CEP68 | -0.133 |
| CEP17-CEP68 | -0.155 |
| CEP18-CEP68 | -0.144 |
| CEP19-CEP68 | -0.155 |
| CEP20-CEP68 | -0.005 |
| CEP21-CEP68 | -0.090 |
| CEP22-CEP68 | -0.113 |
| CEP23-CEP68 | -0.155 |
| CEP24-CEP68 | -0.144 |
| CEP25-CEP68 | -0.096 |
| CEP26-CEP68 | -0.155 |
| CEP27-CEP68 | -0.144 |
| CEP28-CEP68 | -0.036 |
| CEP29-CEP68 | -0.106 |
| CEP30-CEP68 | -0.133 |
| CEP31-CEP68 | -0.117 |
| CEP32-CEP68 | -0.117 |
| CEP33-CEP68 | -0.133 |
| CEP34-CEP68 | -0.090 |
| CEP35-CEP68 | -0.144 |
| CEP36-CEP68 | -0.079 |
| CEP37-CEP68 | -0.144 |
| CEP38-CEP68 | -0.106 |
| CEP39-CEP68 | -0.079 |
| CEP40-CEP68 | 0.103 |
| CEP41-CEP68 | 0.109 |
| CEP42-CEP68 | 0.098 |
| CEP43-CEP68 | 0.123 |
| CEP44-CEP68 | 0.092 |
| CEP45-CEP68 | 0.092 |
| CEP46-CEP68 | 0.211 |
| CEP47-CEP68 | 0.293 |
| CEP48-CEP68 | 0.045 |
| CEP49-CEP68 | 0.066 |
| CEP50-CEP68 | 0.055 |
| CEP51-CEP68 | 0.366 |
| CEP52-CEP68 | 0.333 |
| CEP53-CEP68 | 0.065 |
| CEP54-CEP68 | 0.171 |
| CEP55-CEP68 | 0.089 |
| CEP56-CEP68 | 0.155 |
| CEP57-CEP68 | 0.143 |
| CEP58-CEP68 | 0.018 |
| CEP59-CEP68 | 0.181 |
| CEP60-CEP68 | 0.093 |
| CEP61-CEP68 | 0.123 |
| CEP62-CEP68 | 0.069 |
| CEP63-CEP68 | 0.286 |
| CEP64-CEP68 | 0.120 |
| CEP65-CEP68 | 0.042 |
| CEP66-CEP68 | 0.040 |
| CEP67-CEP68 | 0.092 |
