# Supplementary material for: Take one step backward to move forward: Assessment of genetic diversity and population structure of captive Asian woolly-necked storks (Ciconia episcopus)
Source: PLoS One. 2019 Oct 10;14(10):e0223726. doi: 10.1371/journal.pone.0223726 (PMC6786576; doi:10.1371/journal.pone.0223726)
Supplement: S10 Table — Detailed information for all C. episcopus individuals is presented in S1 Table. (DOCX) [file pone.0223726.s010.docx]

**S10 Table.** Pairwise genetic relatedness (*r*) for all 16 *Ciconia episcopus* individuals in Nakhon Ratchasima Zoo. Detailed information for all *C. episcopus* individuals is presented in S1 Table.

| Individuals pairwise | Pairwise genetic relatedness (*r*) values |
| --- | --- |
| CEP69-CEP70 | -0.144 |
| CEP69-CEP71 | -0.091 |
| CEP70-CEP71 | -0.147 |
| CEP69-CEP72 | -0.144 |
| CEP70-CEP72 | 0.493 |
| CEP71-CEP72 | 0.075 |
| CEP69-CEP73 | 0.129 |
| CEP70-CEP73 | -0.221 |
| CEP71-CEP73 | -0.187 |
| CEP72-CEP73 | -0.221 |
| CEP69-CEP74 | 0.145 |
| CEP70-CEP74 | -0.207 |
| CEP71-CEP74 | -0.110 |
| CEP72-CEP74 | -0.207 |
| CEP73-CEP74 | 0.218 |
| CEP69-CEP75 | -0.138 |
| CEP70-CEP75 | 0.376 |
| CEP71-CEP75 | -0.176 |
| CEP72-CEP75 | 0.287 |
| CEP73-CEP75 | -0.215 |
| CEP74-CEP75 | -0.202 |
| CEP69-CEP76 | 0.029 |
| CEP70-CEP76 | -0.157 |
| CEP71-CEP76 | -0.013 |
| CEP72-CEP76 | -0.157 |
| CEP73-CEP76 | 0.059 |
| CEP74-CEP76 | 0.013 |
| CEP75-CEP76 | -0.152 |
| CEP69-CEP77 | -0.108 |
| CEP70-CEP77 | -0.080 |
| CEP71-CEP77 | 0.005 |
| CEP72-CEP77 | -0.014 |
| CEP73-CEP77 | -0.017 |
| CEP74-CEP77 | -0.040 |
| CEP75-CEP77 | -0.041 |
| CEP76-CEP77 | -0.058 |
| CEP69-CEP78 | 0.057 |
| CEP70-CEP78 | -0.132 |
| CEP71-CEP78 | -0.080 |
| CEP72-CEP78 | -0.132 |
| CEP73-CEP78 | 0.021 |
| CEP74-CEP78 | 0.099 |
| CEP75-CEP78 | -0.127 |
| CEP76-CEP78 | -0.020 |
| CEP77-CEP78 | -0.028 |
| CEP69-CEP79 | 0.090 |
| CEP70-CEP79 | -0.099 |
| CEP71-CEP79 | -0.047 |
| CEP72-CEP79 | -0.099 |
| CEP73-CEP79 | -0.051 |
| CEP74-CEP79 | 0.026 |
| CEP75-CEP79 | -0.094 |
| CEP76-CEP79 | 0.013 |
| CEP77-CEP79 | -0.101 |
| CEP78-CEP79 | 0.101 |
| CEP69-CEP80 | 0.024 |
| CEP70-CEP80 | -0.099 |
| CEP71-CEP80 | -0.147 |
| CEP72-CEP80 | -0.099 |
| CEP73-CEP80 | 0.010 |
| CEP74-CEP80 | 0.023 |
| CEP75-CEP80 | -0.060 |
| CEP76-CEP80 | 0.073 |
| CEP77-CEP80 | 0.031 |
| CEP78-CEP80 | 0.035 |
| CEP79-CEP80 | 0.068 |
| CEP69-CEP81 | -0.083 |
| CEP70-CEP81 | 0.001 |
| CEP71-CEP81 | -0.043 |
| CEP72-CEP81 | -0.088 |
| CEP73-CEP81 | -0.011 |
| CEP74-CEP81 | 0.066 |
| CEP75-CEP81 | -0.028 |
| CEP76-CEP81 | -0.053 |
| CEP77-CEP81 | 0.011 |
| CEP78-CEP81 | 0.033 |
| CEP79-CEP81 | -0.039 |
| CEP80-CEP81 | -0.113 |
| CEP69-CEP82 | 0.090 |
| CEP70-CEP82 | -0.099 |
| CEP71-CEP82 | -0.047 |
| CEP72-CEP82 | -0.099 |
| CEP73-CEP82 | -0.051 |
| CEP74-CEP82 | 0.026 |
| CEP75-CEP82 | -0.094 |
| CEP76-CEP82 | 0.013 |
| CEP77-CEP82 | -0.101 |
| CEP78-CEP82 | 0.101 |
| CEP79-CEP82 | 0.134 |
| CEP80-CEP82 | 0.068 |
| CEP81-CEP82 | -0.039 |
| CEP69-CEP83 | 0.026 |
| CEP70-CEP83 | -0.099 |
| CEP71-CEP83 | 0.001 |
| CEP72-CEP83 | -0.099 |
| CEP73-CEP83 | -0.051 |
| CEP74-CEP83 | -0.037 |
| CEP75-CEP83 | -0.094 |
| CEP76-CEP83 | 0.124 |
| CEP77-CEP83 | -0.101 |
| CEP78-CEP83 | 0.038 |
| CEP79-CEP83 | 0.071 |
| CEP80-CEP83 | 0.068 |
| CEP81-CEP83 | -0.103 |
| CEP82-CEP83 | 0.071 |
| CEP69-CEP84 | -0.151 |
| CEP70-CEP84 | 0.034 |
| CEP71-CEP84 | 0.223 |
| CEP72-CEP84 | -0.055 |
| CEP73-CEP84 | -0.120 |
| CEP74-CEP84 | -0.107 |
| CEP75-CEP84 | 0.005 |
| CEP76-CEP84 | 0.054 |
| CEP77-CEP84 | -0.061 |
| CEP78-CEP84 | -0.140 |
| CEP79-CEP84 | -0.107 |
| CEP80-CEP84 | -0.080 |
| CEP81-CEP84 | 0.139 |
| CEP82-CEP84 | -0.107 |
| CEP83-CEP84 | 0.004 |
