# Supplementary material for: Take one step backward to move forward: Assessment of genetic diversity and population structure of captive Asian woolly-necked storks (Ciconia episcopus)
Source: PLoS One. 2019 Oct 10;14(10):e0223726. doi: 10.1371/journal.pone.0223726 (PMC6786576; doi:10.1371/journal.pone.0223726)
Supplement: S11 Table — Detailed information for all C. episcopus individuals is presented in S1 Table. (DOCX) [file pone.0223726.s011.docx]

**S11 Table.** Pairwise inbreeding coefficients (*F*_IS_) for all 86 *Ciconia episcopus* individuals. Detailed information for all *C. episcopus* individuals is presented in S1 Table.

| Individual | Inbreeding coefficient (*F_IS_*) |
| --- | --- |
| CEP1 | 0.1314 |
| CEP2 | -0.0146 |
| CEP3 | 0.5435 |
| CEP4 | 0.0889 |
| CEP5 | -0.0069 |
| CEP6 | 0.1043 |
| CEP7 | 0.2183 |
| CEP8 | 0.0776 |
| CEP9 | 0.1071 |
| CEP10 | 0.2179 |
| CEP11 | 0.115 |
| CEP12 | 0.7609 |
| CEP13 | 0.1334 |
| CEP14 | -0.0026 |
| CEP15 | 0.0746 |
| CEP16 | 0.0553 |
| CEP17 | 0.1894 |
| CEP18 | 0.0631 |
| CEP19 | 0.2827 |
| CEP20 | 0.0733 |
| CEP21 | 0.0861 |
| CEP22 | 0.5266 |
| CEP23 | 0.1101 |
| CEP24 | 0.0899 |
| CEP25 | -0.0212 |
| CEP26 | -0.0267 |
| CEP27 | 0.259 |
| CEP28 | 0.1321 |
| CEP29 | 0.0786 |
| CEP30 | 0.2435 |
| CEP31 | -0.0126 |
| CEP32 | -0.0263 |
| CEP33 | 0.727 |
| CEP34 | 0.3504 |
| CEP35 | 0.2077 |
| CEP36 | 0.4042 |
| CEP37 | 0.5091 |
| CEP38 | 0.7025 |
| CEP39 | 0.6777 |
| CEP40 | -0.1251 |
| CEP41 | 0.0027 |
| CEP42 | -0.0847 |
| CEP43 | -0.0082 |
| CEP44 | 0.0303 |
| CEP45 | 0.0303 |
| CEP46 | -0.0434 |
| CEP47 | 0.0572 |
| CEP48 | -0.0255 |
| CEP49 | -0.05 |
| CEP50 | -0.0277 |
| CEP51 | 0.1934 |
| CEP52 | 0.1756 |
| CEP53 | -0.0285 |
| CEP54 | -0.0392 |
| CEP55 | 0.2654 |
| CEP56 | 0.0534 |
| CEP57 | -0.0082 |
| CEP58 | -0.0029 |
| CEP59 | 0.6028 |
| CEP60 | -0.1585 |
| CEP61 | 0.0738 |
| CEP62 | -0.1737 |
| CEP63 | 0.1389 |
| CEP64 | -0.0605 |
| CEP65 | -0.0397 |
| CEP66 | 0.0279 |
| CEP67 | -0.124 |
| CEP68 | 0.0794 |
| CEP69 | -0.0387 |
| CEP70 | 0.3625 |
| CEP71 | 0.0441 |
| CEP72 | 0.1575 |
| CEP73 | -0.0581 |
| CEP74 | -0.1898 |
| CEP75 | 0.0192 |
| CEP76 | 0.0121 |
| CEP77 | -0.0291 |
| CEP78 | -0.1632 |
| CEP79 | -0.1322 |
| CEP80 | -0.0057 |
| CEP81 | 0.1578 |
| CEP82 | -0.1322 |
| CEP83 | -0.1293 |
| CEP84 | 0.0836 |
| CEP85 | 0.0885 |
| CEP86 | 0.2266 |
