# Supplementary material for: Take one step backward to move forward: Assessment of genetic diversity and population structure of captive Asian woolly-necked storks (Ciconia episcopus)
Source: PLoS One. 2019 Oct 10;14(10):e0223726. doi: 10.1371/journal.pone.0223726 (PMC6786576; doi:10.1371/journal.pone.0223726)
Supplement: S12 Table — Detailed information for all C. episcopus individuals is presented in S1 Table. (DOCX) [file pone.0223726.s012.docx]

**S12 Table.** Probability of identity using GIMLET version 1.3.3 [40] of *Ciconia episcopus* individuals based on 13 microsatellite loci. Detailed information for all *C. episcopus* individuals is presented in S1 Table.

| Locus | Unbias./loc. | Prod (unbias.) |
| --- | --- | --- |
| Wsu13 | 6.12E-01 | 6.12E-01 |
| Cc10 | 1.25E-01 | 7.62E-02 |
| Ah211 | 1.57E-01 | 1.19E-02 |
| Cc02 | 5.40E-01 | 6.44E-03 |
| Cc06 | 5.78E-01 | 3.72E-03 |
| Cc42 | 4.13E-01 | 1.54E-03 |
| Cbo121 | 8.85E-02 | 1.36E-04 |
| Cc07 | 9.40E-02 | 1.28E-05 |
| Cbo109 | 1.34E-01 | 1.71E-06 |
| Cc04 | 1.39E-01 | 2.37E-07 |
| Cbo151 | 3.00E-01 | 7.11E-08 |
| Cbo108 | 4.32E-01 | 3.07E-08 |
| Cc37 | 1.00E+00 | 3.07E-08 |
| Mean | 3.55E-01 | 5.48E-02 |
| S.D. | 0.275 | 0.169 |
