# Supplementary material for: Take one step backward to move forward: Assessment of genetic diversity and population structure of captive Asian woolly-necked storks (Ciconia episcopus)
Source: PLoS One. 2019 Oct 10;14(10):e0223726. doi: 10.1371/journal.pone.0223726 (PMC6786576; doi:10.1371/journal.pone.0223726)
Supplement: S13 Table — The number indicates p values, with 110 permutations. Detailed information for all C. episcopus individuals is presented in S1 Table. (DOCX) [file pone.0223726.s013.docx]

**S13 Table.** Pairwise genetic differentiation (*F_ST_*), pairwise *F*_ST_^ENA^ values with ENA correction for null alleles and *R*_ST_ values using FSTAT version 2.9.3 [30] and of *Ciconia episcopus* between captive breeding based on 13 microsatellite loci. The number indicates *p* values, with 110 permutations. Detailed information for all *C. episcopus* individuals is presented in S1 Table.

| Combination | *F*_ST_ | *F*_ST_^ENA^ | *R*_ST_ |
| --- | --- | --- | --- |
| Khao Kheow Open Zoo x Nakhon Ratchasima Zoo | 0.15027* | 0.090931 | 0.16283 |
| Khao Kheow Open Zoo x Dusit Zoo | 0.18686* | 0.085223 | 0.20685 |
| Nakhon Ratchasima Zoo x Dusit Zoo | 0.14352* | 0.010671 | 0.15492 |

**p* value < 0.05
