# Supplementary material for: Take one step backward to move forward: Assessment of genetic diversity and population structure of captive Asian woolly-necked storks (Ciconia episcopus)
Source: PLoS One. 2019 Oct 10;14(10):e0223726. doi: 10.1371/journal.pone.0223726 (PMC6786576; doi:10.1371/journal.pone.0223726)
Supplement: S14 Table — Detailed information for all C. episcopus individuals is presented in S1 Table. (DOCX) [file pone.0223726.s014.docx]

**S14 Table.** Analysis of molecular variance (AMOVA) results of *Ciconia episcopus* based on 13 microsatellite loci using Arlequin version 3.5.2.2 [27]. Detailed information for all *C. episcopus* individuals is presented in S1 Table.

| Source of variation | df | Sum of squares | Variance components | Percentage of variation |
| --- | --- | --- | --- | --- |
| Among populations | 2 | 37.601 | 0.54244 | 15.51 |
| Within populations | 169 | 499.574 | 2.95606 | 84.49 |
| Total | 171 | 537.174 | 3.4985 |  |
