# Supplementary material for: Take one step backward to move forward: Assessment of genetic diversity and population structure of captive Asian woolly-necked storks (Ciconia episcopus)
Source: PLoS One. 2019 Oct 10;14(10):e0223726. doi: 10.1371/journal.pone.0223726 (PMC6786576; doi:10.1371/journal.pone.0223726)
Supplement: S15 Table — (DOCX) [file pone.0223726.s015.docx]

**S15 Table.** Pairwise population Nei’s genetic distance (*D*) values using GenAlEx version 6.5 [31] of 86 *Ciconia episcopus* individuals in each zoo based on 13 microsatellite loci**.**

| *Nei D* | Khao Kheow Open Zoo | Nakhon Ratchasima Zoo | Dusit Zoo |
| --- | --- | --- | --- |
| Khao Kheow Open Zoo | 0.000000 |  |  |
| Nakhon Ratchasima Zoo | 0.159591 | 0.000000 |  |
| Dusit Zoo | 0.284589 | 0.160942 | 0.000000 |
