# Supplementary material for: Take one step backward to move forward: Assessment of genetic diversity and population structure of captive Asian woolly-necked storks (Ciconia episcopus)
Source: PLoS One. 2019 Oct 10;14(10):e0223726. doi: 10.1371/journal.pone.0223726 (PMC6786576; doi:10.1371/journal.pone.0223726)
Supplement: S16 Table — Detailed information for all C. episcopus individuals is presented in S1 Table. (DOCX) [file pone.0223726.s016.docx]

**S16 Table.** Genetic bottlenecks of *Ciconia episcopus* individuals in the three zoos using BOTTLENECK version 1.2.02 [43] and calculation of *M* ratio using Arlequin version 3.5.2.2 [27] for all populations. Detailed information for all *C. episcopus* individuals is presented in S1 Table.

| Species | Captivity/wild | Wilcoxon signed-rank test | | Mode-shift test | *M ratio* |
| --- | --- | --- | --- | --- | --- |
|  |  | SMM | TPM |  |  |
| *Ciconia episcopus* | Khao Kheow Open Zoo | 0.997 | 0.455 | L-shaped mode | 0.292 |
|  | Nakhon Ratchasima Zoo | 0.080 | 0.005 | shifted mode | 0.254 |
|  | Dusit Zoo | N/A | N/A | N/A | 0.278 |

“N/A”: Not available.
